# Range dynamics of *Anopheles* mosquitoes in Africa suggest a significant increase in the malaria transmission risk

**DOI:** 10.1002/ece3.70059

**Published:** 2024-07-31

**Authors:** Peixiao Nie, Chunyan He, Jianmeng Feng

**Affiliations:** ^1^ College of Agriculture and Biological Science Dali University Dali China; ^2^ Cangshan Forest Ecosystem Observation and Research Station of Yunnan Province Dali University Dali China

**Keywords:** Africa, *Anopheles* species, future scenarios, malaria transmission, range dynamics

## Abstract

Despite a more than 100‐year effort to combat malaria, it remains one of the most malignant infectious diseases globally, especially in Africa. Malaria is transmitted by several *Anopheles* mosquitoes. However, until now few studies have investigated future range dynamics of major *An*. mosquitoes in Africa through a unified scheme. Through a unified scheme, we developed 21 species distribution models to predict the range dynamics of 21 major *An*. species in Africa under future scenarios and also examined their overall range dynamic patterns mainly through suitability overlap index and range overlap index. Although future range dynamics varied substantially among the 21 *An*. species, we predicted large future range expansions for all 21 *An*. species, and increases in suitability overlap index were detected in more than 90% of the African continent for all future scenarios. Additionally, we predicted high range overlap index in West Africa, East Africa, South Sudan, Angola, and the Democratic Republic of the Congo under future scenarios. Although the relative impacts of land use, topography and climate variables on the range dynamics depended on species and spatial scale, climate played the strongest roles in the range dynamics of most species. Africa might face an increasing risk of malaria transmissions in the future, and better strategies are required to address this problem. Mitigating climate change and human disturbance of natural ecosystems might be essential to reduce the proliferation of *An*. species and the risk of malaria transmissions in Africa in the future. Our strategies against their impacts should be species‐specific.

## Graphical abstract

We used a unified scheme and developed 105 species distribution models to examine the range dynamics of as many as 21 major *An*. species in Africa through several novel ecological indicators. Although the range ratio indices differed substantially among the species, the ranges of all species were predicted to increase in the future. Additionally, increases in the suitability overlap index of all 21 *An*. species was predicted for more than 90% of the African continent. Our results suggested that Africa might face increasing threats of malaria epidemics in the future.

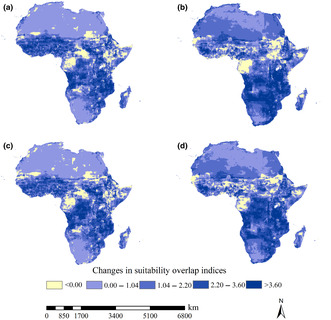

## 1 INTRODUCTION

Although more than 100 years have been spent trying to combat malaria, it remains one of the most malignant infectious diseases worldwide (Hay et al., 2004; Nájera et al., 2011; Okumu et al., 2022; World Health Organization, 2023). Approximately 250 million malaria cases and 600,000 malaria deaths have annually occurred in the world in recent decades (World Health Organization, 2023). The disease is a major problem in tropical regions, especially in Africa, posing a substantial threat to people in Africa (Semakula et al., 2017; World Health Organization, 2023). Malaria is a vector‐transmitted disease; therefore, vector control is an effective countermeasure in Africa (Amadi et al., 2018; Benelli & Beier, 2017; Sherrard‐Smith et al., 2022; Sougoufara et al., 2020; Tawe et al., 2017). The detection of hotspots is required for efficient vector control. Therefore, predicting distribution patterns or potential ranges of malaria vectors (i.e., *Anopheles* mosquitoes) has attracted much attention in recent years. Most studies focused on several major malaria vectors, e.g., *An. gambiae* complex, and *An. funestus* (Adeogun et al., 2023; Mataba et al., 2023; Mboera et al., 2015; Mmbando et al., 2021). However, few studies have investigated the future range dynamics of all major *An*. species in Africa through a unified scheme, and thereby detected their overall priority regions and compared their risks. For example, although both Akpan et al. (2019) Olabimi et al. (2021) and projected the future range dynamics of *Anopheles gambiae* in Africa, they adopted different predictors, spatial scales and future scenarios, which, to a certain extent, rendered the two studies not comparable.

Climate change has been expected to influence the ranges of malaria vectors, i.e., *An*. mosquitoes (Carlson et al., 2023; Li et al., 2021; Olabimi et al., 2021) because climate conditions strongly affect the development and life history of *An*. mosquitoes (e.g., Agyekum et al., 2021; Oliver & Brooke, 2017; Zengenene et al., 2021). Although the range dynamics in *An*. mosquitoes in Africa in the future have received much attention (e.g., Akpan et al., 2019; Alimi et al., 2015; Ryan et al., 2023), substantial controversy remains. For example, Drake and Beier (2014) projected a range loss of *An. arabiensis* in Africa in the future, which was supported by the findings of Peterson (2009). In contrast, Alimi et al. (2015) detected the opposite patterns of range shifts of *An*. mosquitoes. Additionally, Olabimi et al. (2021) projected that the range dynamics of *An. gambiae* in Southwest Nigeria in the future depended on the general circulation models (GCMs) used to project climate conditions in the future, i.e., range contraction or expansions. Therefore, range shifts of *An*. mosquitoes in the future require more in‐depth study.

In addition to climate change, anthropogenic habitat disturbance, primarily land‐use change, could significantly affect range shifts in *An*. mosquitoes because land‐use change might modify *An*. mosquito habitat (Omukunda et al., 2012). An increase in cultivated land (farmland) and a decrease in natural land cover generally cause range expansions of *An*. mosquitoes. For example, an increase in rain‐fed cropland and a decrease in natural vegetation cover might cause range expansions of *An*. mosquitoes, increasing the risk of malaria cases in sub‐Saharan Africa (Shah et al., 2022). Moreover, Omukunda et al. (2012) detected range expansions of *An. gambiae* and *An. funestus* in Western Kenya due to swamp cultivation. Although anthropogenic habitat disturbance might substantially affect the potential ranges of malaria vectors, its influences relative to climate change remain controversial. For example, climate change had a larger influence on the range shifts of dominant malaria vector species in Nigeria than land‐use change (Akpan et al., 2019). This finding was supported by Adeogun et al. (2023). However, Ageep et al. (2009) detected stronger influences of land use on the range shifts of *An. arabiensis* in northern Sudan than climate change. Therefore, the relative influences of land use change and climate change on the range dynamics in *An*. mosquitoes require further investigations.

Topographical factors (slope, aspect, and elevation) might represent barriers to species dispersal (Virkkala et al., 2010) and can substantially modify the spatial allocations of energy and water, resulting in various habitats (Dudov, 2017; Luoto & Heikkinen, 2008; Tang et al., 2018). Although it is well known that climate change and anthropogenic disturbances significantly affect the range of *An*. mosquitoes in Africa, the influences of topography should not be overlooked. For example, Adeogun et al. (2023) detected the impacts of topographical factors on the ranges of *An. coluzzii*, *An. gambiae*, and *An. arabiensis* in Nigeria. Additionally, Mwakalinga et al. (2018) argued that topographical factors might be responsible for the major variation in the distribution of malaria vectors in Tanzania. However, the effects of topographical factors relative to climate are unknown. For example, Gwitira et al. (2015) detected stronger influences of topographical variables than of climate predictors on the ranges of *An. arabiensis* in Zimbabwe, which was supported by the findings by Tanga et al. (2010) and Alimi et al. (2015). However, the observations of Adeogun et al. (2023) showed opposite patterns. Thus, the relative influences of topography and climate on the ranges of *An*. mosquitoes require additional research.

The present study aimed to use a unified scheme to examine future range dynamics of major *An*. mosquitoes in Africa through adopting unified spatial scale, future scenarios, candidate predictors and candidate algorithms, et al. We hypothesized that the range dynamics of *An*. mosquitoes and the relative influences of topography, land use and climate on the range dynamics might depend on the species. Therefore, we used topography, land use and climate data to assess the range dynamics of 21 *An*. species in Africa. We hope our study can provide meaningful information for devising strategies to prevent the spread of malaria vectors.

## 2 METHODS

### 2.1 Occurrences of *An*. Mosquitoes in Africa

**FIGURE 1:**
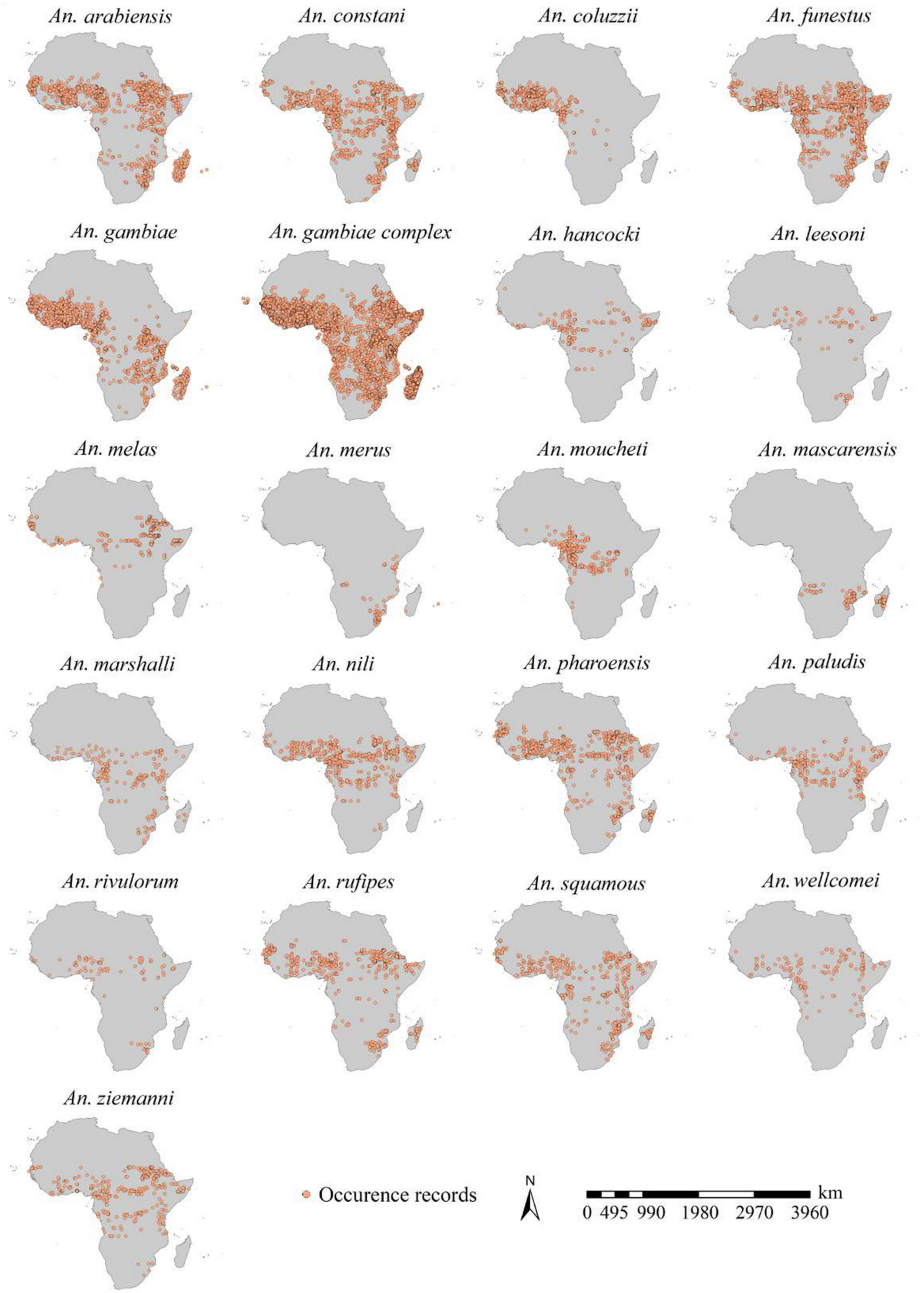
Occurrences of 21 *Anopheles* species in Africa. Records were compiled from the Global Biodiversity Information Facility (GBIF) and a published compendium by Kyalo et al. (2016). There was a total of 22,716 occurrence records after spatial rarefication.

From a published compendium by Kyalo et al. (2016), we retrieved the initial list of 23 *An*. species in Africa (Figure 1). There were 23 items of *An*. species in this compendium. In our study, we combined *An*. *funestus* s.l and *An*. *funestus* s.s. into a species, i.e., *An*. *funestus*. We also incorporated savannah or Bamako forms of *An. gambiae* into *An. gambiae*. Therefore, we totally obtained 21 *An*. species in Africa. We used two sources to determine the occurrences of *An*. mosquitoes in Africa, i.e., a published compendium of occurrence data of malaria vectors in Africa (Kyalo et al., 2016) and the Global Biodiversity Information Facility (GBIF) database, a widely accepted comprehensive online dataset of global species occurrence records containing more than 2.6 billion records from approximately 2100 publishing institutions, 90,000 datasets, and 9300 publications. We retrieved 39,848 records for *An*. mosquitoes in Africa from the published compendium of occurrence data (Kyalo et al., 2016) and 154,989 records from the GBIF database. Then, we generated a preliminary occurrence dataset for each species, totally including 194,837 records of 21 *An*. species. As suggested by Nie and Feng (2023), we only retained occurrence records with an uncertainty of geographical coordinates of less than 5 km. We considered the sampling bias effects and thinned the occurrences with a 5 km radius for each *An*. species individually (Brown et al., 2017; Liu et al., 2020). We finally built a final occurrence dataset for each species, totally containing 22,714 records of *An*. mosquitoes (Figure 1, Data S1).

### 2.2 Predictors for species distribution model

We used 30 factors to predict the potential ranges and habitat suitability of each *An*. species individually, including climate (19), land use (8), and topographical (3) factors. The spatial resolution of the 19 climatic variables was 2.5 arc‐min. They included eight precipitation and 11 temperature predictors (Fick & Hijmans, 2017). The climate predictors for the current and future scenarios were retrieved from Worldclim (Fick & Hijmans, 2017). The current climatic predictors were extracted from the climatic datasets for the near‐current period in Worldclim (Fick & Hijmans, 2017). The climate variables used for the future scenario in 2100 were calibrated using the global climate models (GCMs) FIO‐ESM‐2‐0 (F) and MPI‐ESM1‐2‐HR (M), the two most robust and complementary GCMs (Zhang et al., 2022). We adopted two Shared Socio‐Economic Pathways (SSPs) in 2100, i.e., SSP126 (126) and SSP585 (585), representing the most optimistic and pessimistic future climate change scenarios in 2100, in this order. Totally, five sets of climatic predictors were created, i.e., predictors under current scenario, predictors under SSP126 scenario calibrated by the GCM FIO‐ESM‐2‐0 (F126), predictors under SSP585 scenario calibrated by the GCM FIO‐ESM‐2‐0 (F585), predictors under SSP126 scenario calibrated by the GCM MPI‐ESM1‐2‐HR (M126), and predictors under SSP585 scenario calibrated by the GCM MPI‐ESM1‐2‐HR (M585). Therefore, for each species' potential ranges, we had five scenarios, i.e., current, F126, F585, M126, and M585 scenarios.

Eight land use types under current and future scenarios were obtained from the Land‐Use Harmonization dataset (LUH2) (https://luh.umd.edu/) with a spatial resolution of 0.25 arc degree: rangeland, non‐forested primary land, forested primary land, managed pasture, forested secondary land, cropland, urban land, and non‐forested secondary land fractions. There were three scenarios for land use datasets, i.e., current scenario, SSP126 scenario in 2100 and SSP585 scenario in 2100. The source data of topographical factors (i.e., slope, elevation, and aspect), i.e., digital elevation model (DEM), were retrieved from Worldclim with 0.5 arc‐min‐spatial‐resolution (ca. 1 km) (Fick & Hijmans, 2017). We extracted elevation from the DEM and calculated slope and aspect on the basis of the DEM at the 0.5 arc‐min resolution. Then, we used majority method to resample elevation, slope and aspect to a coarser resolution of 2.5 arc‐min, i.e., determining the value of each pixel based on the most popular value within a 3 by 3 window. The topographical predictors remain unchanged under five scenarios. The spatial data of the factors were or resampled to a resolution of 2.5 arc‐min.

### 2.3 Selection of predictors

We created preliminary species distribution models (SDMs) for each species individually through Biomod2, an ensemble platform for species distribution modeling (Thuiller et al., 2009), and used jackknife technique to determine each predictor's importance which were represented by its importance value outputted by the preliminary SDMs (Data S2). We used 10 algorithms: Flexible Discriminant Analysis, Artificial Neural Network, Multiple Adaptive Regression Splines, Generalized Linear Model, Classification Tree Analysis, Generalized Boosting Model, Random Forest for Classification and Regression, Maximum Entropy Model, Surface Range Envelope and EXtreme Gradient Boosting (Thuiller et al., 2009). We used following methods to downscale all predictors, i.e., bioclimatic, land‐use and topographical variables. Firstly, Pearson's correlation analyses were performed to determine the collinearity between predictor pairs using |.7| as the threshold (Nie & Feng, 2023) (Data S3). Then, if high collinearity was identified between a pair, the predictor with the lower IV was removed. The retained variables were input into the final SDMs to project the species' maps of habitat suitability index and potential ranges for each species individually, as well as the importance value of each predictor.

### 2.4 Predicting habitat suitability and potential ranges

For each species individually, we predicted the maps of habitat suitability index and ranges of the *An*. species using Biomod2, an ensemble platform for species distribution modeling (Thuiller et al., 2009), and the 10 candidate algorithms used in our preliminary SDMs were adopted (*R* code available at https://doi.org/10.7910/DVN/HYI36S). As recommended by Thuiller et al. (2009), a three‐fold random selection was conducted to determine pseudo‐absences (PAs) across African continent. If the number of occurrences of the *An*. species was <1000, 1000 PAs were randomly generated; otherwise, the amount of PAs was equal to that of the *An*. species (Barbet‐Massin et al., 2012). We created initial habitat suitability index maps of each *An*. species, individually. Then, we adopted the maximum sensitivity–specificity (MSS) approach (Liu et al., 2016) to transform habitat suitability index maps of each *An*. species into binary values of potential/no‐potential ranges of each *An*. species, individually.

For each species individually, we adopted five‐fold cross‐validation to evaluate the SDMs' performances. We randomly selected 70% of the records to train the SDMs and the remaining 30% to assess the SDMs's reliability (Yang et al., 2021). For each species individually, we removed algorithms with true skill statistic (TSS) of less than 0.6 and area under the curve (AUC) of less than 0.8 (Della Rocca & Milanesi, 2022; Nie & Feng, 2023). Therefore, algorithms used in the final SDMs were species‐specific (Data S4). To obtain assembled SDM projection, we gave each model's projection a weight proportional to their TSS evaluation. In total, we developed 21 baseline SDMs to project habitat suitability index and potential ranges of the 21 *An*. species under current scenarios. The predictors under future scenarios were inputted into 21 baseline SDMs to predict future ranges for each species, individually.

### 2.5 Predicting shifts in habitat suitability and potential ranges

The habitat suitability index shifts for each *An*. species were individually estimated by subtracting the map of habitat suitability indices under the future scenario from the map under the current scenario. We also built and calibrated suitability overlap index (SOI) (i.e., $\mathrm{SOI}=\sum_{i=1}^{N=21}{\mathrm{HSI}}_{i}$) maps through overlap habitat suitability maps of the 21 *An*. species under each scenario individually, in which HSI was the habitat suitability index. Similarly, we obtained the maps of suitability overlap index shifts for the 21 *An*. species by subtracting the maps of suitability overlap indices under the future scenario from the maps under the current scenario. Finally, as suggested by Chen et al. (2013), we used Natural Breaks (Jenks) approach to categorize maps of suitability overlap index and the changes in suitability overlap index. We divided the potential ranges of each species into three categories: expanding ranges (ER), unfilling ranges (UR) and stabilizing ranges (SR), which represented ranges only occupied under in the future, those occupied only in the current period, and those occupied under current and future scenarios, respectively (Nie & Feng, 2023). Accordingly, the ranges under the current situation (RC) were the total of the SR and UR, and those under the future scenarios (RF) were the sum of ER and SR (Nie & Feng, 2023). The shifts in potential ranges for the *An*. species were assessed using the range ratio (RR) and range similarity (RS) (Nie & Feng, 2023). The RR was used to compare the sizes of the RC and RF:

$$\mathrm{RR}=\frac{\mathrm{RF}}{\mathrm{RC}},$$

If RR < 1, RF is smaller than RC.

The RS was estimated to investigated the changes in range positions for the *An*. species from the current to the future scenarios:

$$\mathrm{RS}=\frac{2\text{OR}}{\mathrm{RC}+\mathrm{RF}},$$

where OR is the ranges shared by the current and future scenarios for each species. If RS < 0.5, *An*. mosquito under future and current scenarios potentially occupied different range positions.

We also created and calibrated maps of range overlap index (ROI), which was rendered as follows:

$$\mathrm{ROI}=\sum_{i=1}^{N=21}{\mathrm{PR}}_{i},$$

in which PR_*i*_ was the potential ranges of each species. For each scenario individually, we retrieved the range overlap index maps through overlapping the potential ranges of all 21 *An*. species, and assessed their spatial patterns. Through similar methods, we also obtained maps of expanding range overlap index (EROI), unfilling range overlap index (UROI) and stabilizing range overlap index (SROI) of the 21 species for each scenario individually, and examined their spatial patterns.

## 3 RESULTS

### 3.1 Reliability of the species distribution models

The AUC scores of the species distribution models for the 21 *An*. species ranged from 0.937 to 0.992, with an average of 0.966 ± 0.016. The TSSs ranged from 0.715 to 0.940, with an average of 0.819 ± 0.061 (Data S5). The high values of the AUC scores and TSSs indicated the robustness of the SDMs and the projected maps of habitat suitability and potential ranges of each *An*. species in Africa. Specifically, the highest AUCs (ca. 0.990) were detected in *An. merus* (0.992) and *An. mascarensis* (0.991), while the lowest AUCs were identified in *An. funestus* (0.948), *An. squamous* (0.945), *An. nili* (0.940) and *An. gambiae* complex (0.937) (Data S5). The highest TSSs (>0.900) were detected in *An. merus* (0.940) and *An. mascarensis* (0.934), while the lowest ones (<0.750) were mainly identified in *An. squamous* (0.733), *An. gambiae* complex (0.718) and *An. nili* (0.715) (Data S5).

### 3.2 Relative influences of predictors

**FIGURE 2:**
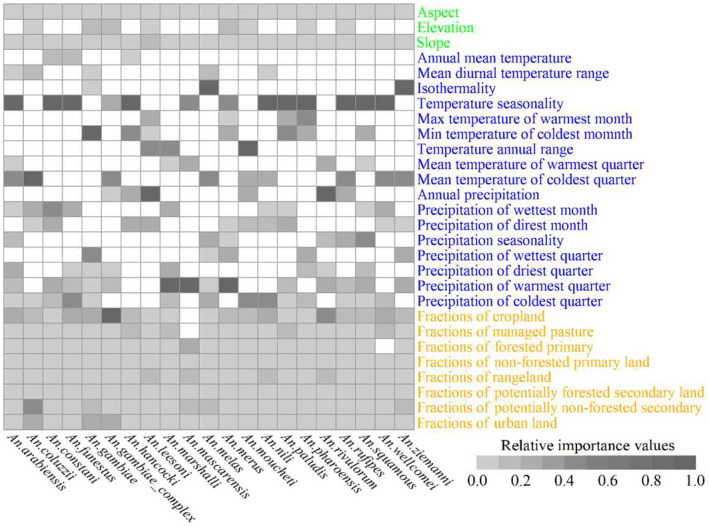
Importance of the predictors in the species distribution models. Topographic, climatic and human disturbance predictors were in green, blue and yellow, respectively. We min‐max standardized the importance values of the predictors for each species, separately. The blanks indicated that predictors were not in our final and formal models.

The top predictors of the potential ranges differed for the different *An*. species (Figure 2, Data S6). For example, the top predictors of the ranges of *An. gambiae* were the minimum temperature in the coldest month (important value of 0.084), precipitation in the wettest season (0.081), and cropland (0.070) (Figure 2, Data S6). The top predictors for the ranges of *An. arabiensis* were temperature seasonality (0.113), mean temperature in winter (0.091), and cropland (0.068) (Figure 2, Data S6). The highest importance value (IV) (0.610) was detected in precipitation in the warmest season in the SDMs for *An. mascarensis*, while the lowest ones (ca. zero) varied with species (Figure 2, Data S6). In summary, the climatic predictors had the highest important values for the ranges of 20 *An*. species, followed by land‐use type and topography (Figure 2, Data S6). In contrast, the top predictors for the *An. gambiae* complex were land use factors, followed by climate and topographical factors (Figure 2, Data S6). Additionally, the importance values of the topographical predictors were lower than those of the climate and anthropogenic disturbance predictors in most SDMs (Figure 2, Data S6).

### 3.3 Habitat suitability of *An*. Mosquitoes in Africa

The spatial patterns of habitat suitability indices varied with scenarios. For example, *An. mascarensis* under the F126 scenario was projected to show high habitat suitability index in Angola and the southeastern part of Africa (Data S7), while under the M585 scenario, its high habitat suitability index was mostly projected in Central African Republic, Angola, Democratic Republic of the Congo, and the southeastern part of Africa (Data S7). The spatial patterns of habitat suitability indices were also specific. For example, *An. gambiae* under the F585 scenario was projected to have high habitat suitability index in East Africa and tropical regions of Africa, while for *An. melas* under this scenario, its high habitat suitability index was mostly detected in tropical regions of Africa (Data S7).

**FIGURE 3:**
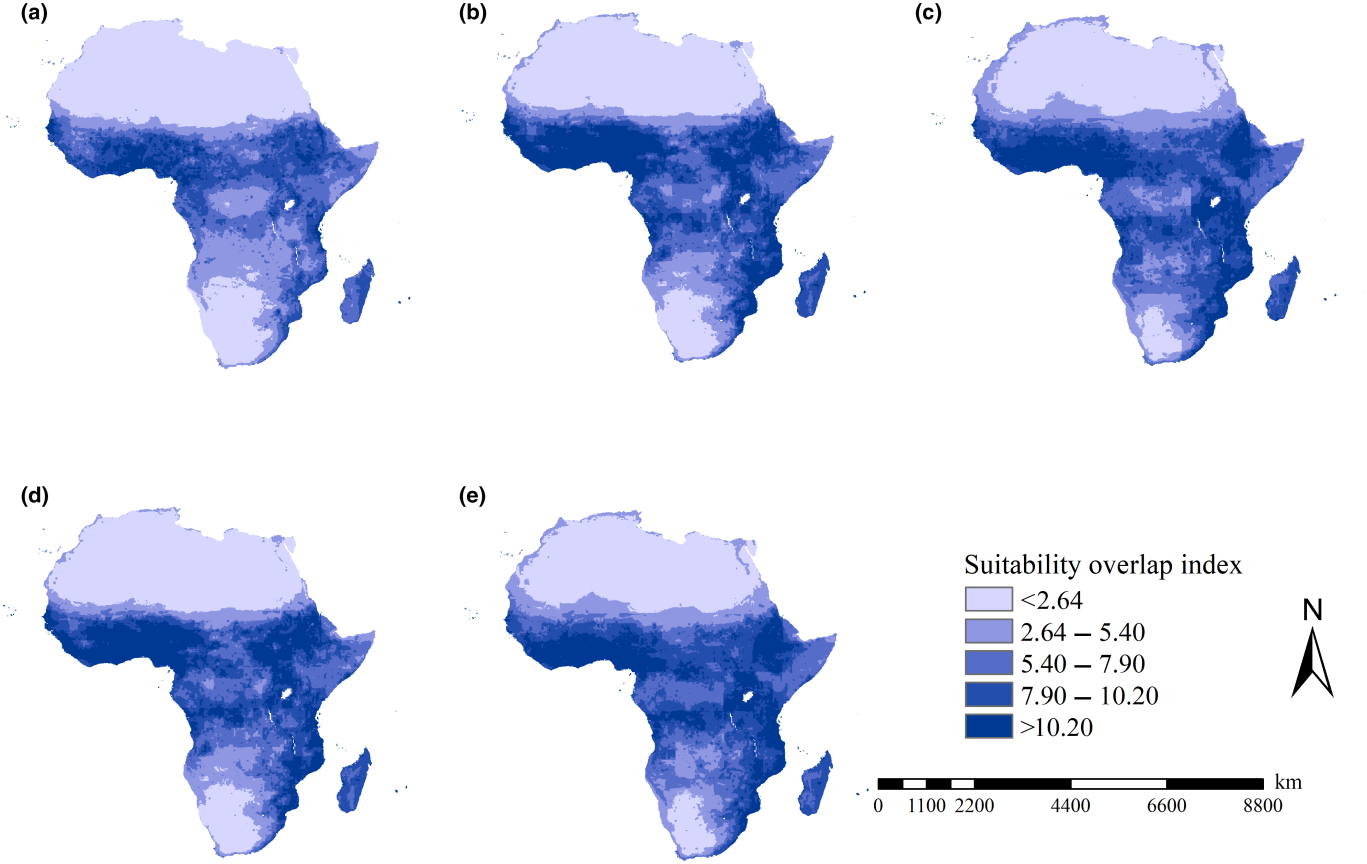
Suitability overlap indices of 21 *Anopheles* species in Africa. (a) Current conditions; (b) F126 scenario; (c) F585 scenario; (d) M126 scenario; (e) M585 scenario. Under all scenarios, high suitability overlap indices were mainly detected in the regions with latitude from equator to 15 north latitude degree in Africa, and it was also identified in Tanzania, Mozambique, Swaziland and Madagascar.

The spatial patterns of suitability overlap indices of the 21 *An*. species differed for different scenarios. Under the current and F126 scenarios, high suitability overlap indices were projected to occur in the southeastern part of Africa and the regions from the equator to 15° north latitude (Figure 3). However, under the M126, F585 and M585 scenarios, high suitability overlap indices were mostly projected in the regions from the equator to 15° north latitude, most part of East Africa and Angola.

**FIGURE 4:**
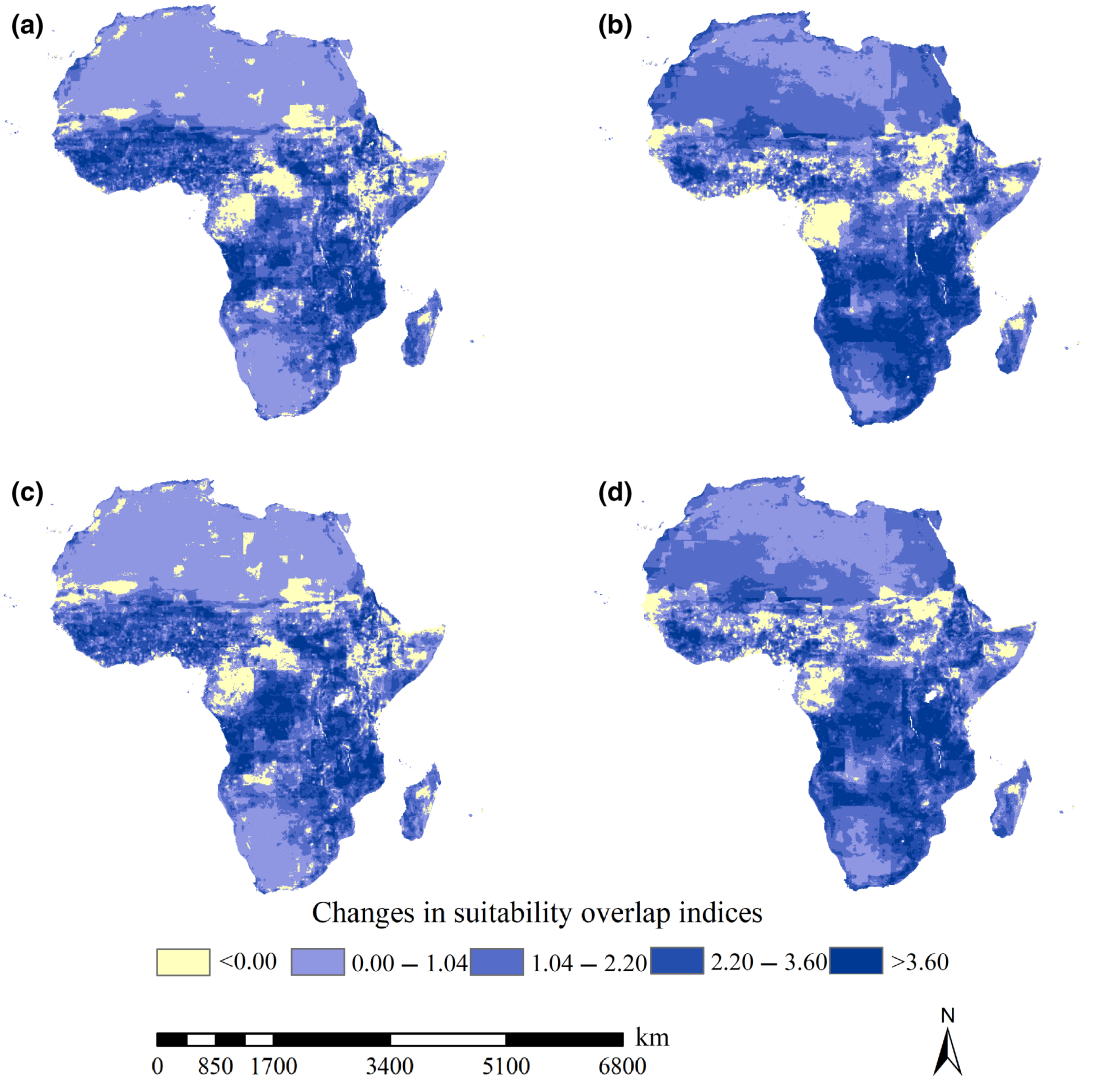
Changes in suitability overlap index of 21 *Anopheles* species under current‐future scenarios in Africa. (a) Current‐F126; (b) current‐F585; (c) current‐M126; (d) current‐M585. Substantial increases in suitability overlap indices were mainly projected in the southern part of West Africa, the southeastern part of Africa and Central Africa. Under the current‐F585 and current‐M585 scenarios, substantial increases in suitability overlap indices were primarily detected in the southeastern part of Africa, Central Africa and the scattered regions in southern part of West Africa.

Under the F126 and M126 scenarios, substantial increases in suitability overlap indices were predicted in the southern part of West Africa, the southeastern part of Africa and Central Africa, while substantial decreases in suitability overlap indices were identified in the Central African Republic, Republic of the Congo, Cameroon, and Gabon (Figure 4). Under the current‐F585 and current‐M585 scenarios, substantial increases in suitability overlap indices were detected in the southeastern part of Africa, Central Africa and the scattered regions in southern part of West Africa. Also, under the current‐F585 and current‐M585 scenarios, considerable decreases in suitability overlap indices were mostly detected in Gabon, Central Africa, South Sudan, Sudan, and Somalia Senegal (Figure 4). Additionally, areas with decreases in suitability overlap indices covered 2.58, 2.61, 2.90, and 2.28 million km^2^ under the current‐F126, current‐F585, current‐M126, and current‐M585 scenarios, respectively. Areas with increases in suitability overlap indices covered 27.62, 27.59, 27.29, and 27.91 million km^2^. Therefore, more than ca. 90% of the African continent exhibited increases in suitability overlap indices for the 21 *An*. mosquitoes under current and future scenarios.

### 3.4 Potential ranges of *An.* Species in Africa

The MSS values differed for different *An*. species and scenarios (Data S8). For example, under current conditions, the MSS values were 0.450 and 0.620 for *An*. *gambiae* and *An. arabiensis* and 0.400 and 0.240 for *An. melas* in the F126 and F585 scenarios, respectively. The MSS values ranged from 0.110 to 0.770 (Data S8). The highest MSS thresholds were primarily projected in *An. gambiae* (0.77 under F585 scenario), *An. gambiae* complex (0.76 under F126 scenario) and *An. gambiae* complex (0.74 under M126 scenario), while the lowest ones were mainly detected in *An. merus* (0.11, 0.14 and 0.15 under F585, M585 and F126 scenarios, respectively) (Data S8).

The spatial patterns of the potential ranges differed for different *An*. species (Data S9). For example, the current ranges of *An. hancocki* was mainly in the western part of Central Africa, and Somalia, covering 2.12 million km^2^ (Table 1, Data S9), while those of *An. mascarensis* was in Angola, Zambia, and the southeastern part of Africa, covering 2.34 million km^2^ (Table 1, Data S9). The spatial patterns of the potential ranges also varied with scenarios (Table 1, Data S9). For example, the range of *An. coluzzii* under the current scenario was mostly projected in tropical regions of West Africa, covering 2.42 million km^2^, while those under the F585 scenario was mostly projected in tropical regions of Africa, Angola, East Africa, and covered 7.13 million km^2^ (Table 1, Data S9).

**TABLE 1:** Potential ranges and range dynamics of 21 *Anopheles* mosquitoes under current and future scenarios.

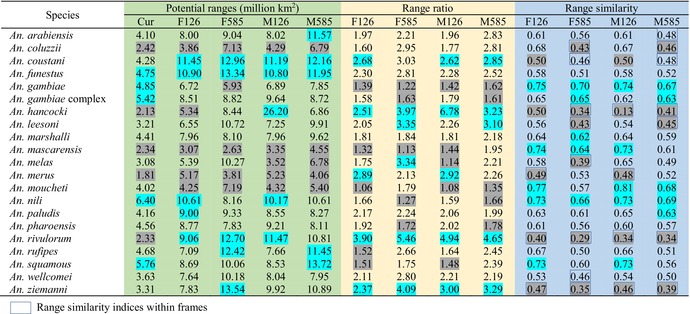

*Note*: Cur, current scenarios; F126, F126 scenarios; F585, F585 scenarios; M126, M126 scenarios; M585, M585 scenarios; The light green, light yellow and light blue backgrounds indicated the areas of potential ranges, range ratio indices and range similarity indices, respectively. The blue highlighted indicated the top five largest potential ranges, the top five highest range ratio indices and top five highest range similarity indices under each scenario. The gray highlighted indicated the five smallest potential ranges, lowest range ratio indices and lowest range similarity indices under each scenario. Range similarity indices within frames indicated the potential ranges under current and future scenarios occupied different positions.

The sizes of the potential range varied with species and scenarios. For example, under the current scenarios, we identified the largest potential range in *An. nili* (6.40 million km^2^), and smallest one in *An. merus* (1.81 million km^2^) (Table 1). Under the F126 scenarios, the potential ranges of *An. mascarensis* covered 3.07 million km^2^, while under the F585 scenarios, it covered 2.63 million km^2^ (Table 1).

Accordingly, the areas covered by the potential ranges of 21 *An*. species ranged from 1.81 to 6.40, from 3.07 to 11.45, from 2.63 to 13.54, from 3.35 to 26.20 and from 4.06 to 13.72 million km^2^ under current, F126, F585, M126 and M585 scenarios, respectively (Table 1). Paired sample *t*‐test showed that the areas of the current potential ranges of all 21 target species were smaller than those under future scenarios (*p* < .01). *An. funestus*, *An. coustani* and *An. nili*, and *An. rivulorum* occurred five, four, three, and three times in the top five largest potential ranges under all scenarios, respectively (Table 1), while *An. coluzzii*, *An. mascarensis*, *An. merus*, and *An. moucheti* occurred five, five, five, and four times in the five smallest potential ranges under all scenarios (Table 1).

**FIGURE 5:**
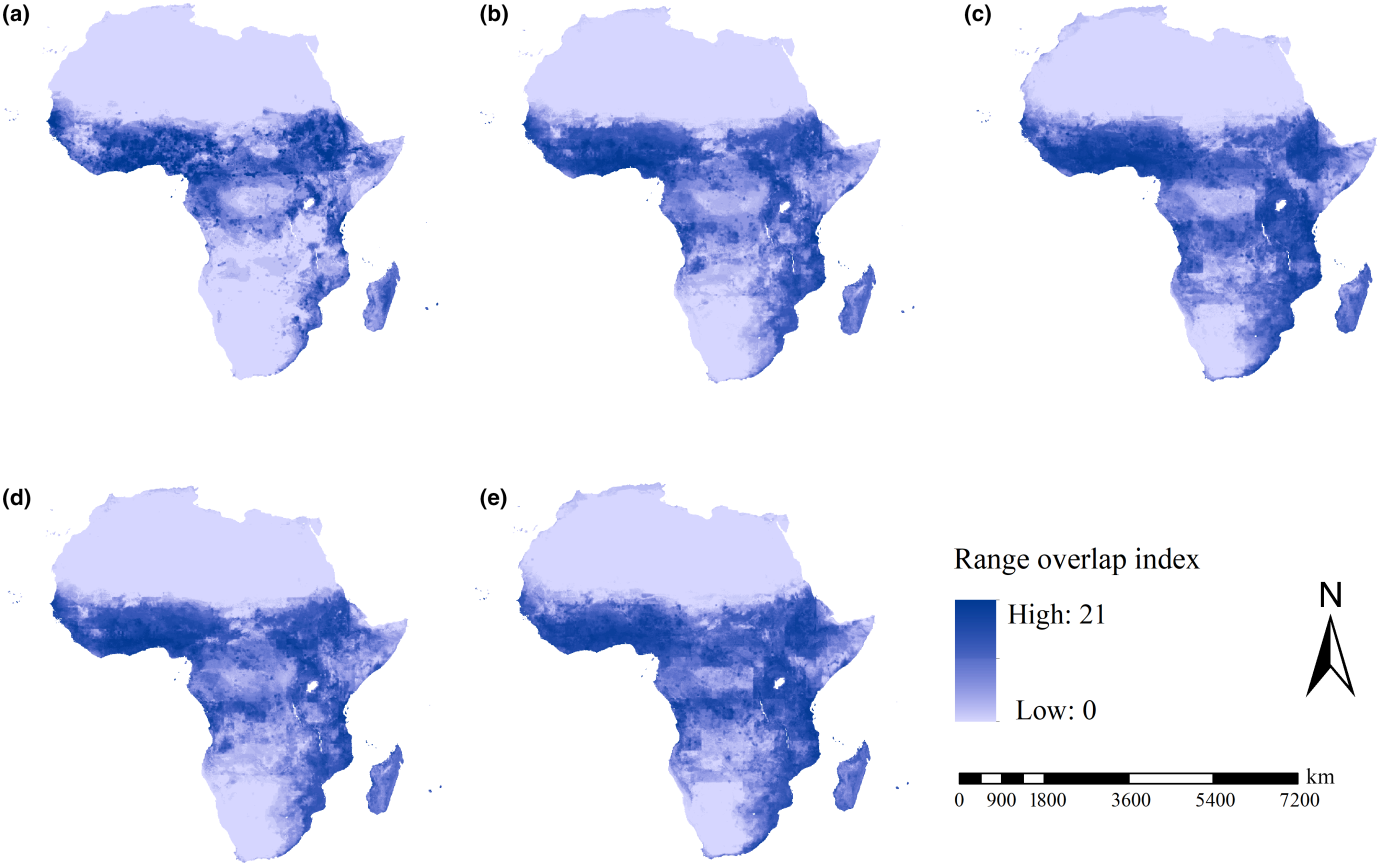
Range overlap indices of 21 *Anopheles* species. (a) Range overlap indices under current conditions; (b) range overlap indices under F126 scenario; (c) range overlap indices under F585 scenario; (d) range overlap indices under M126 scenario; (e) range overlap indices under M585 scenario. Under all scenarios, high range overlap indices were detected in West Africa and East Africa, South Sudan, Angola and Democratic Republic Congo.

Although the spatial patterns of range overlap indices varied with scenarios, the regions from the equator to 15° north latitude the high range overlap indices of the all 21 *An*. species under future scenarios were mostly detected in the southeastern part of Africa and the regions from the equator to 15° north latitude (Figure 5).

### 3.5 Range dynamics of the *An*. Species in Africa

The range dynamics also differed for different *An*. species and scenarios (Table 1). For example, under current‐F126 scenario, range ratios in *An. rivulorum* and *An. gambiae* were 3.90 and 1.39, respectively, while under current‐F585 scenario scenarios, the range ratios were 5.46 and 1.22, respectively (Table 1). Under current‐F126 scenario, range similarity for *An. merus* and *An. melas* was 0.49 and 0.58, respectively, while under current‐M585 scenarios, the range similarity was 0.52 and 0.49, respectively. Under the F126 scenarios, the range ratios ranged from 1.06 in *An. moucheti* to 3.90 in *An. rivulorum*. *An. hancocki*, *An. rivulorum*, *An. ziemanni*, and *An. coustani*, occurred four, four, four, and three times, respectively, in the lists of the top five highest range ratios under the four future scenarios (Table 1). *An. hancocki*, *An. rivulorum*, and *An. ziemanni* were detected four times in the lists of the five lowest range similarity (largest shifts in range centroids) under four future scenarios (Table 1).

All *An*. species were projected to show range expansions under all future scenarios (i.e., all range ratios >1, Table 1). The averages of range ratios were 2.00, 2.29, 2.54, and 2.42 under F126, M126, F585 and M585 scenarios, respectively (Table 1). Most of range similarity for 21 *An*. species under four future scenarios were higher than 0.5 (Table 1). The averages of range similarity were 0.61, 0.59, 0.52 and 0.53 under F126, M126, F585 and M585 scenarios, respectively.

**FIGURE 6:**
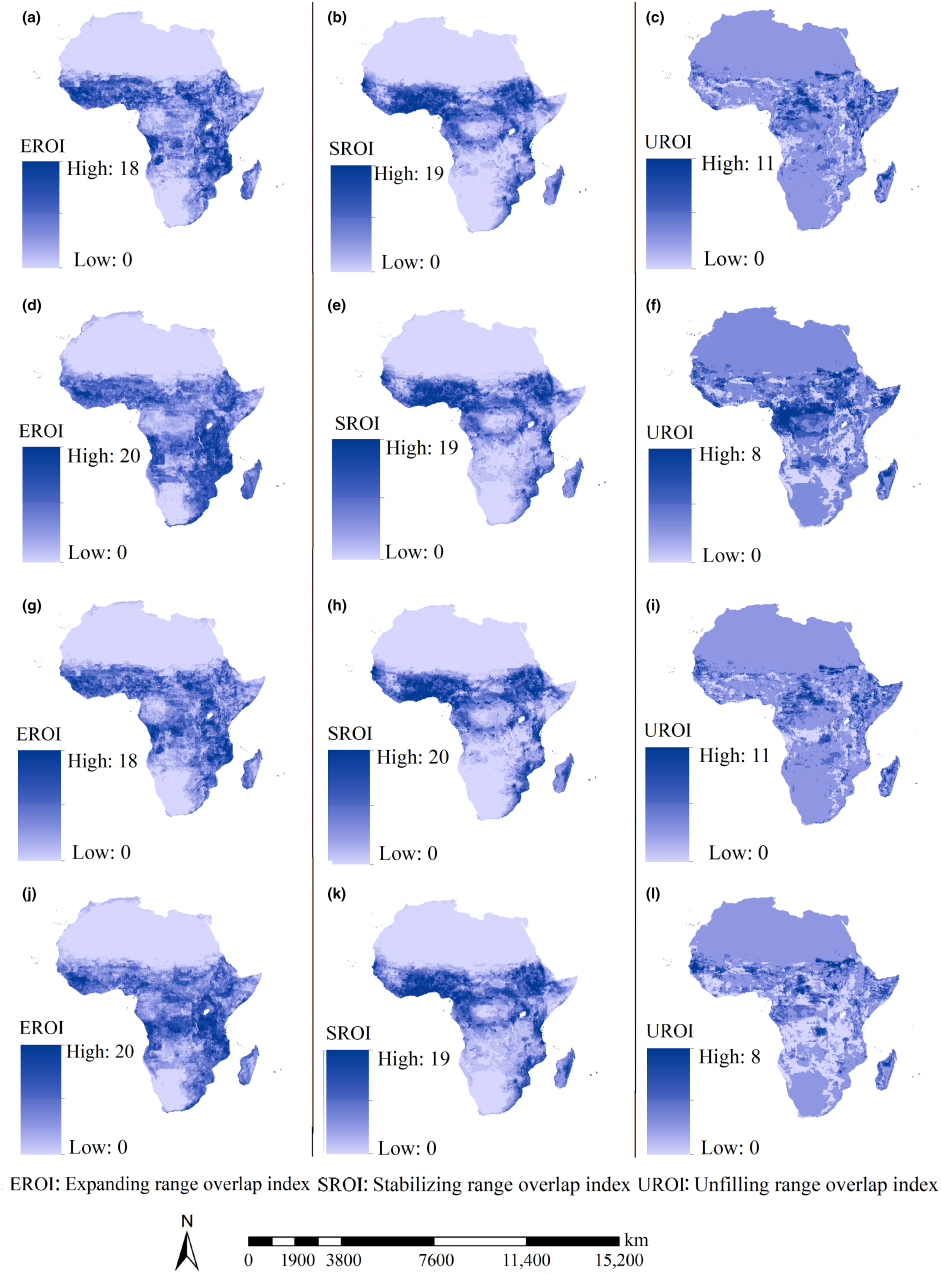
Overlapped expanding, stabilizing and unfilling ranges of 21 *Anopheles* species under current‐future scenarios. (a) Expanding range overlap index under current‐F126 scenarios; (b) stabilizing range overlap index under current‐F126 scenarios; (c) unfilling range overlap index under current‐F126 scenarios; (d) expanding range overlap index under current‐F585 scenarios; (e) stabilizing range overlap index under current‐F585 scenarios; (f) unfilling range overlap index under current‐F585 scenarios; (g) expanding range overlap index under current‐M126 scenarios; (h) stabilizing range overlap index under current‐M126 scenarios; (i) unfilling range overlap index under current‐M126 scenarios; (j) expanding range overlap index under current‐M585 scenarios; (k) stabilizing range overlap index under current‐M585 scenarios; (l) unfilling range overlap index under current‐M585 scenarios.

The high expanding range overlap indices of 21 *An*. mosquitoes under all current‐future scenarios were mostly projected to scattered in the regions from the equator to 15° north latitude, Central Africa and the southeastern part of Africa (Figure 6). The high unfilling range overlap indices under all current‐future scenarios which might be potentially occupied by the *An. mosquitoes* under current conditions might lose in the future scenarios were mostly projected to scatter in Central African (Figure 6). The high stabilizing range overlap indices under all future scenarios were mainly detected in east coastline of Africa and the regions with latitude from equator to 15 north latitude except South Sudan (Figure 6).

## 4 DISCUSSION

We used two robust and complementary GCMs and established 21 SDMs to project the range dynamics of 21 *An*. species in Africa mainly through suitability overlap index and range overlap index. Our results indicated range expansions of all 21 *An*. species under current and future scenarios. Additionally, increases in the suitability overlap indices of 21 *An*. species were predicted for more than 90% of the African continent. Therefore, Africa might face an increasing malaria risk in the future, and more effective strategies will be needed to combat malaria. Our results are consistent with the findings of Tanser et al. (2003) and Caminade et al. (2014), although they used different methodologies and target species. Diouf et al. (2022) used the Liverpool malaria model (LMM), a mathematical‐biological dynamic malaria model, to simulate the vectors' population dynamics, and predicted that the impacts of future climate changes on malaria in West Africa (the dominant region of the *An*. species ranges in our study) might decrease in the future. These contradictory results imply that further studies should be conducted on the prediction of future malaria transmissions in Africa. Additionally, through a unified scheme, we investigated the range dynamics of the 21 *An*. species, and hereby made the comparisons of their range dynamics possible. We also elucidated the range stabilizing, range expanding, and range unfilling of the 21 *An*. species. Therefore, our study could provide novel and essential information for developing future strategies against their impacts.

Many studies have shown that climate change and anthropogenic habitat disturbance (such as land use modifications) strongly affect the ranges of *An*. species (e.g., Adeogun et al., 2023; Ageep et al., 2009; Akpan et al., 2018; Shah et al., 2022). However, their relative roles in the range dynamics remain unclear. For example, Ageep et al. (2009) detected stronger influences of land use than climate on the range shifts of *An*. species. Adeogun et al. (2023) detected the opposite pattern. Sirami et al. (2017) insisted that the factors' relative influences on range dynamics depended on the scale, i.e., climate change had a stronger role at a larger scale, whereas land‐use change might have larger effects at a smaller scale. Our continental‐scale study showed that climate had larger effects than land use on the ranges of 20 *An*. species in Africa. These results are supported by the findings of Alimi et al. (2015), Akpan et al. (2018), Akpan et al. (2019) and Sirami et al. (2017). Our results also showed that land use had stronger influences than climate on the range of the *An. gambiae* complex, consistent with the findings of Srivastava et al. (2013). These observations suggested that the relative influences of land use and climate on their range dynamics not only depend on the spatial scale but are species‐specific. For example, Mataba et al. (2023) argued that distribution of *An. gambiae* complex in Tanzania was positively affected by its proximity to the land use types with high human population density, probably due to its they mainly feed on humans indoor. However, the relatively stronger influences of climate on the potential ranges of *An. arabiensis* in our study were probably associated with its tendency to feed on animals other than humans (Lindsay et al., 1998), suggesting weak effects of human‐dominated land‐use types on its distribution.

Several studies demonstrated that topographical factors substantially affected the ranges of *An*. species (e.g., Alimi et al., 2015; Mwakalinga et al., 2018; Wanjala & Kweka, 2016). Others indicated that the relative roles of topographical and climate factors remained unclear (e.g., Adeogun et al., 2023; Alimi et al., 2015). Additionally, some studies found that elevation (the most frequently investigated topographical factor) had a larger influence on range dynamics than climatic factors, although the study areas were smaller than ours. For example, Tanga et al. (2010) found that the distribution of *An*. species in southwestern Cameroon were largely determined by elevation. A study in Zimbabwe produced similar results (Gwitira et al., 2015). However, our continental‐scale study indicated that topographical variables had smaller influences than climate factors on the ranges of all 21 *An*. species. These findings imply that the relative influences of climate and topography on the range dynamics of *An*. species in Africa might depend on the spatial scale. A possible reason is that elevation is a comprehensive proxy of climate conditions (such as temperature and humidity) and has a large influence at a small scale, whereas the same does not hold at a large scale. Another possible explanation is that our coarse spatial resolution (ca. 1 km) of topographical predictors could not identify many smaller larval habitats of *An*. species. Our study showed that climate change exhibited substantial effects on the range dynamics of most *An*. species in Africa. Therefore, future climate changes might result in range expansions, potentially increasing the risk of malaria transmission. Several studies had the same conclusion (e.g., Ebi et al., 2005; Haines & Patz, 2004; Le et al., 2019; Paaijmans et al., 2010). Therefore, mitigating future climate change is critical to prevent malaria transmission in Africa. Our study also predicted that future land‐use change, especially an increase in the proportion of cropland, contributed to the range expansion of the *An. gambiae* complex, one of the essential vectors responsible for malaria transmission, suggesting that land‐use change might increase the risk of malaria transmission. These findings aligned with those of Omukunda et al. (2012) and Kweka et al. (2016). Therefore, the conversion of natural land to cropland in Africa should be minimized due to the potential effect on *An*. species range expansion and increased malaria risk (Akogbéto et al., 2018; Gillies & Coetzee, 1987; Gillies & De Meillon, 1968; Sinka et al., 2012).

Senegal, Gambia, Ivory Coast, Sierra Leone, Nigeria, Ghana, South Sudan, Ethiopia, Uganda, Democratic Republic of the Congo, Zambia, Malawi, Angola, Zimbabwe, Tanzania, and Mozambique are hotspots for malaria transmission in Africa (World Health Organization, 2023). Our predictions showed that the suitability overlap indices for *An*. species might increase substantially there in the future. Therefore, more effective strategies are required to prevent or mitigate malaria transmission. Our study suggested substantial range expansions of *An*. species to occur in scattered regions of Angola, Guinea, Ghana, Nigeria, South Sudan, Kenya, Tanzania, and Mozambique. Although the proliferation of *An*. species and the malaria epidemic risk are currently low in these scattered regions, we should implement better strategies to prevent or minimize the range expansions to reduce the risk of future malaria transmission in these regions.

Our study predicted substantial variations in the range dynamics of 21 *An*. species. This finding suggested that different strategies should be used for *An*. species with different range dynamics. Additionally, under most future scenarios *An. funestus*, *An. coustani*, *An. nili*, and *An. rivulorum* were projected to have larger potential ranges than others, suggesting that more attention should be paid on them in the future. *An. hancocki*, *An. rivulorum*, *An. ziemanni*, *An. coustani*, and *An. merus* were projected to show larger range ratios than others under most future scenarios, implying their substantial range expansions in the future. Therefore, much stricter strategies should be devised for them. *An. hancocki*, *An. rivulorum*, and *An. ziemanni* exhibited lower range similarity than other *An*. species in all future scenarios, suggesting substantial shifts in their range positions. This finding suggests that the current priority regions for reducing their proliferation might not be the same in the future, and substantial strategy modification may be required.

## 5 CONCLUSIONS

We used two robust and complementary GCMs to predict the range dynamics of 21 *An*. species through 21 species distribution models. To our best knowledge, this is the first report in which the range dynamics of as many as 21 *An*. species were examined. Although the range dynamics differed substantially among the species, the ranges of all species were predicted to increase in the future. Additionally, increases in the habitat suitability of all 21 *An*. species was predicted for more than 90% of the African continent. Our results suggested that Africa might face increasing threats of malaria transmission in the future. Therefore, more effective strategies will be needed to combat future malaria transmission. Mitigating climate change and anthropogenic disturbances of natural ecosystems due to cropland conversion will be essential to prevent or mitigate the proliferation of *An*. species and the risk of malaria transmission in Africa.

## AUTHOR CONTRIBUTIONS

**Peixiao Nie:** Formal analysis (lead); investigation (lead); resources (lead); software (lead); validation (lead); visualization (lead); writing – original draft (equal); writing – review and editing (equal). **Chunyan He:** Formal analysis (supporting); investigation (supporting); resources (supporting); software (supporting); validation (supporting); visualization (supporting); writing – original draft (supporting); writing – review and editing (supporting). **Jianmeng Feng:** Conceptualization (lead); data curation (lead); funding acquisition (lead); methodology (lead); project administration (lead); supervision (lead); writing – original draft (equal); writing – review and editing (equal).

## CONFLICT OF INTEREST STATEMENT

The authors declare that they have no known competing financial interests or personal relationships that could have appeared to influence the work reported in this paper.

## Supporting information


Data S1:

Data S2:

Data S3:

Data S4:

Data S5:

Data S6:

Data S7:

Data S8:

Data S9:

## Data Availability

The data and materials underlying this article are available in the article and in its Supporting Information.
