# Supplementary material for: Range dynamics of Anopheles mosquitoes in Africa suggest a significant increase in the malaria transmission risk
Source: Ecol Evol. 2024 Jul 31;14(8):e70059. doi: 10.1002/ece3.70059 (PMC11289791; doi:10.1002/ece3.70059)
Supplement: Supplementary file 5 — Data S5: [file ECE3-14-e70059-s002.docx]

| Species Name | The number of occurrences after spatial rarefication | AUC | TSS |
| --- | --- | --- | --- |
| *Anopheles gambiae* | 3090 | 0.967 | 0.802 |
| *Anopheles arabiensis* | 2101 | 0.960 | 0.795 |
| *Anopheles melas* | 395 | 0.981 | 0.856 |
| *Anopheles merus* | 90 | 0.992 | 0.940 |
| *Anopheles funestus* | 2291 | 0.948 | 0.763 |
| *Anopheles rivulorum* | 89 | 0.970 | 0.828 |
| *Anopheles leesoni* | 88 | 0.979 | 0.874 |
| *Anopheles nili* | 639 | 0.940 | 0.715 |
| *Anopheles moucheti* | 394 | 0.978 | 0.852 |
| *Anopheles pharoensis* | 1096 | 0.962 | 0.792 |
| *Anopheles hancocki* | 202 | 0.969 | 0.848 |
| *Anopheles mascarensis* | 197 | 0.991 | 0.934 |
| *Anopheles marshalli* | 209 | 0.959 | 0.799 |
| *Anopheles squamous* | 582 | 0.945 | 0.733 |
| *Anopheles wellcomei* | 162 | 0.969 | 0.836 |
| *Anopheles rufipes* | 609 | 0.955 | 0.776 |
| *Anopheles constani* | 1467 | 0.980 | 0.862 |
| *Anopheles ziemanni* | 426 | 0.968 | 0.817 |
| *Anopheles paludis* | 281 | 0.958 | 0.774 |
| *Anopheles coluzzii* | 783 | 0.987 | 0.884 |
| *Anopheles gambiae_complex* | 7523 | 0.937 | 0.718 |

S5 AUCs and TSSs in the final species distribution models
