# Supplementary material for: Range dynamics of Anopheles mosquitoes in Africa suggest a significant increase in the malaria transmission risk
Source: Ecol Evol. 2024 Jul 31;14(8):e70059. doi: 10.1002/ece3.70059 (PMC11289791; doi:10.1002/ece3.70059)
Supplement: Supplementary file 7 — Data S7: [file ECE3-14-e70059-s001.pdf]

S7 Maps of habitat suitability index of 21 *Anopheles* species

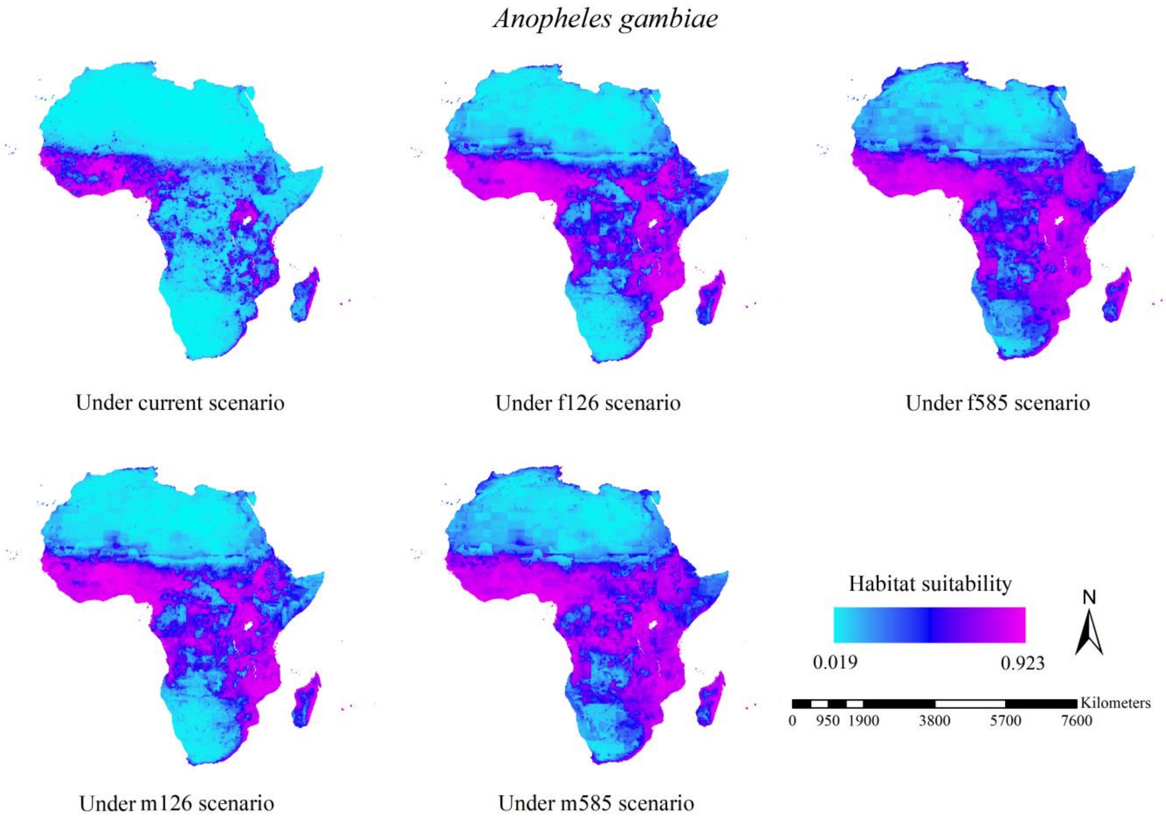

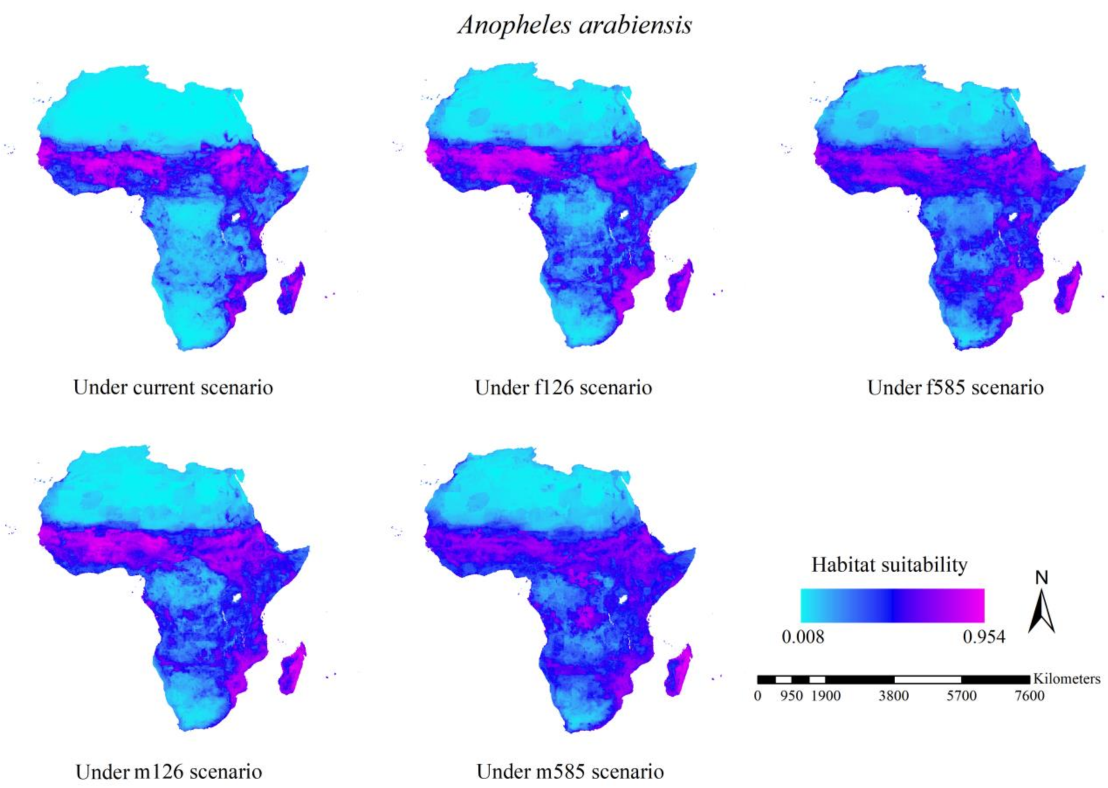
Anopheles arabiensis
Under current scenario
Under f126 scenario
Under f585 scenario
Under m126 scenario
Under m585 scenario
Habitat suitability
0.008
0.954
N
Kilometers
0 950 1900 3800 5700 7600

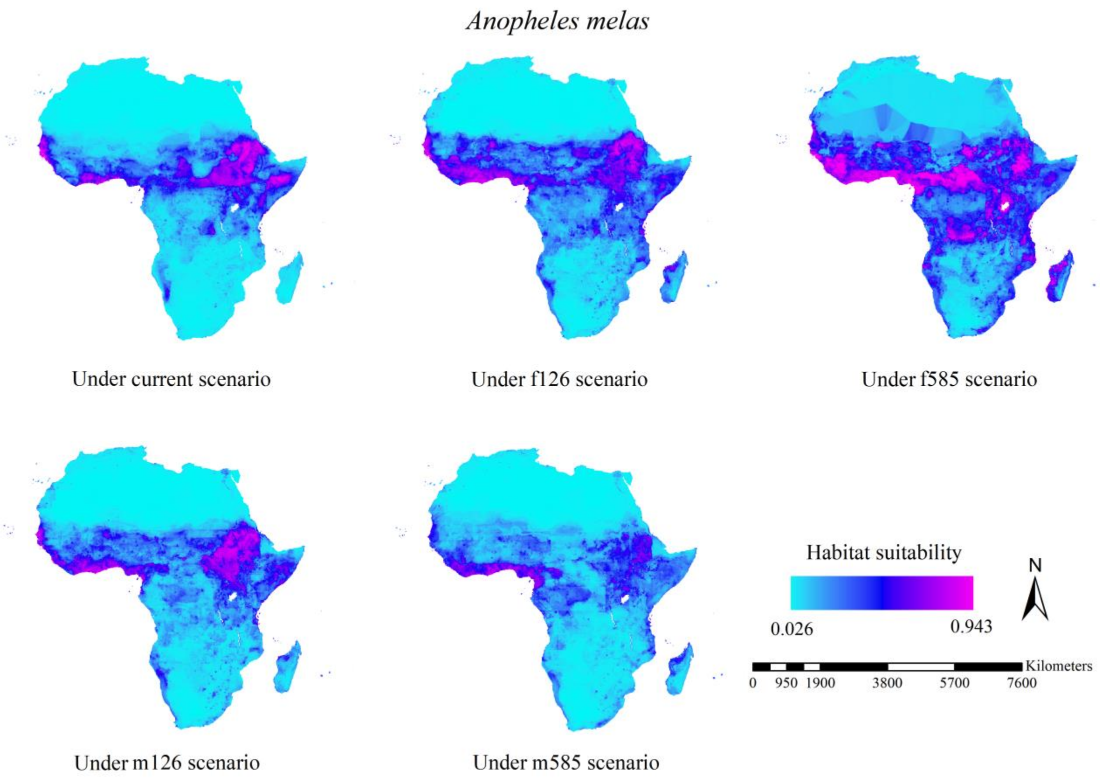
Anopheles melas
Under current scenario
Under f126 scenario
Under f585 scenario
Under m126 scenario
Under m585 scenario
Habitat suitability
0.026
0.943
N
Kilometers
0 950 1900 3800 5700 7600

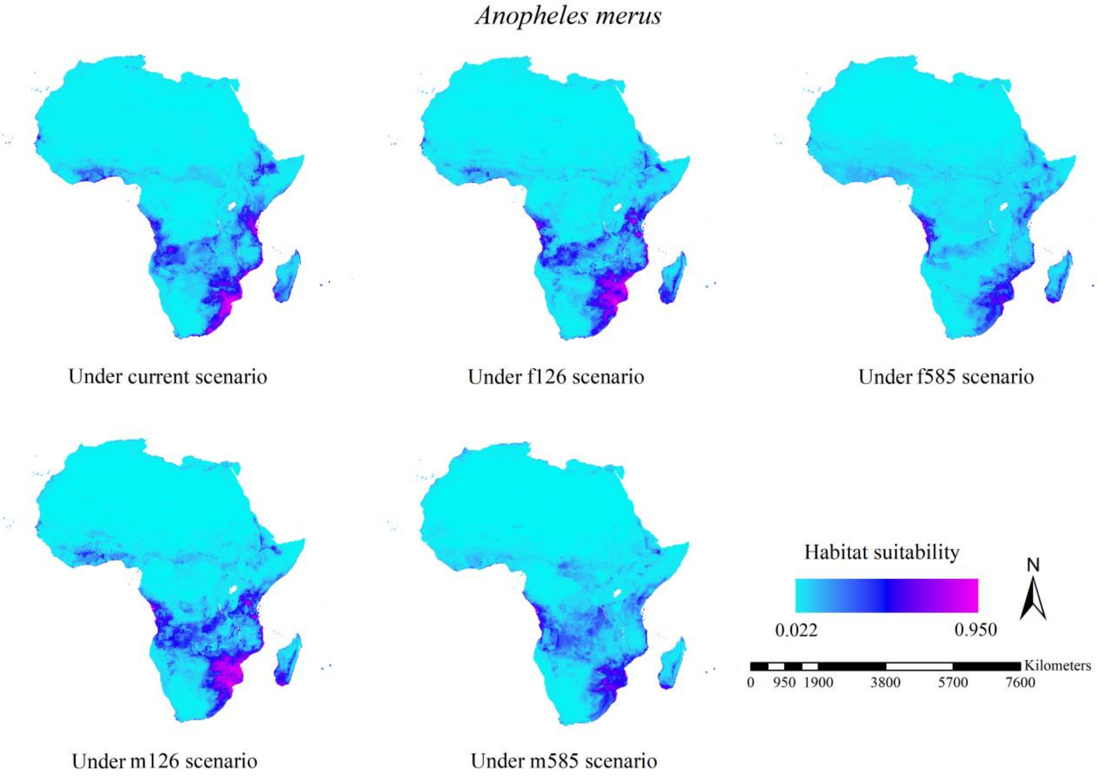
Anopheles merus
Under current scenario
Under f126 scenario
Under f585 scenario
Under m126 scenario
Under m585 scenario
Habitat suitability
0.022
0.950
N
Kilometers
0
950
1900
3800
5700
7600

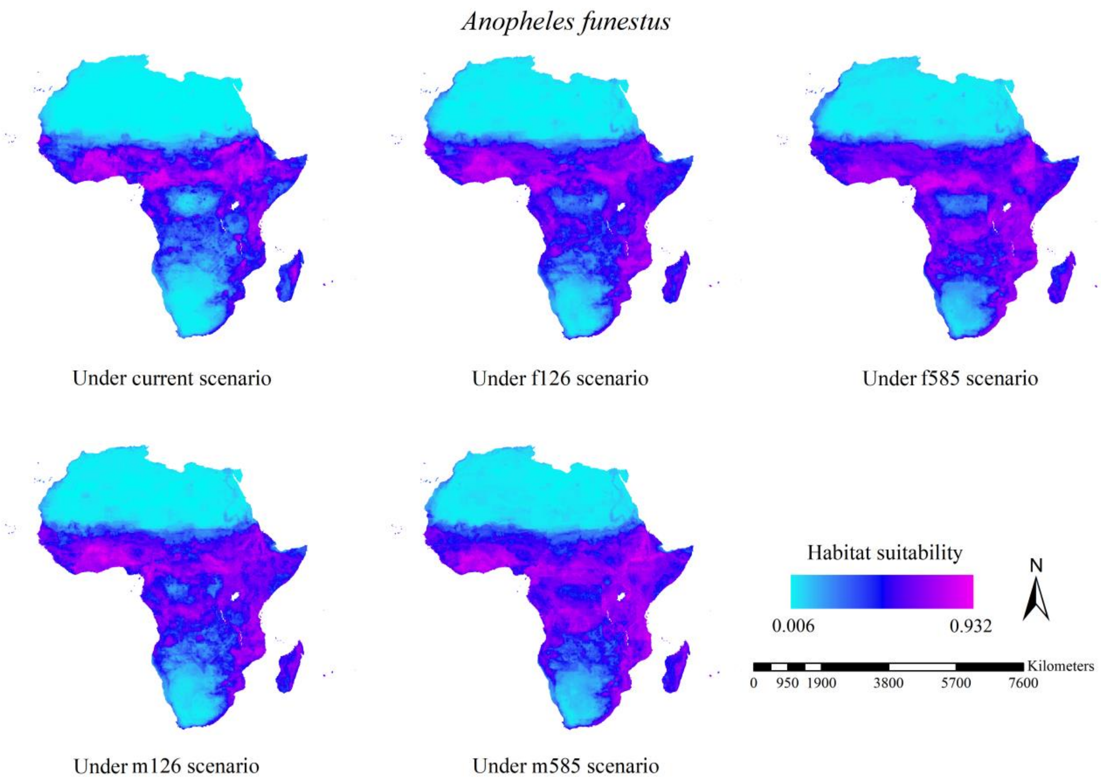
Anopheles funestus
Under current scenario
Under f126 scenario
Under f585 scenario
Under m126 scenario
Under m585 scenario
Habitat suitability
0.006
0.932
N
Kilometers
0 950 1900 3800 5700 7600

Anopheles rivulorum

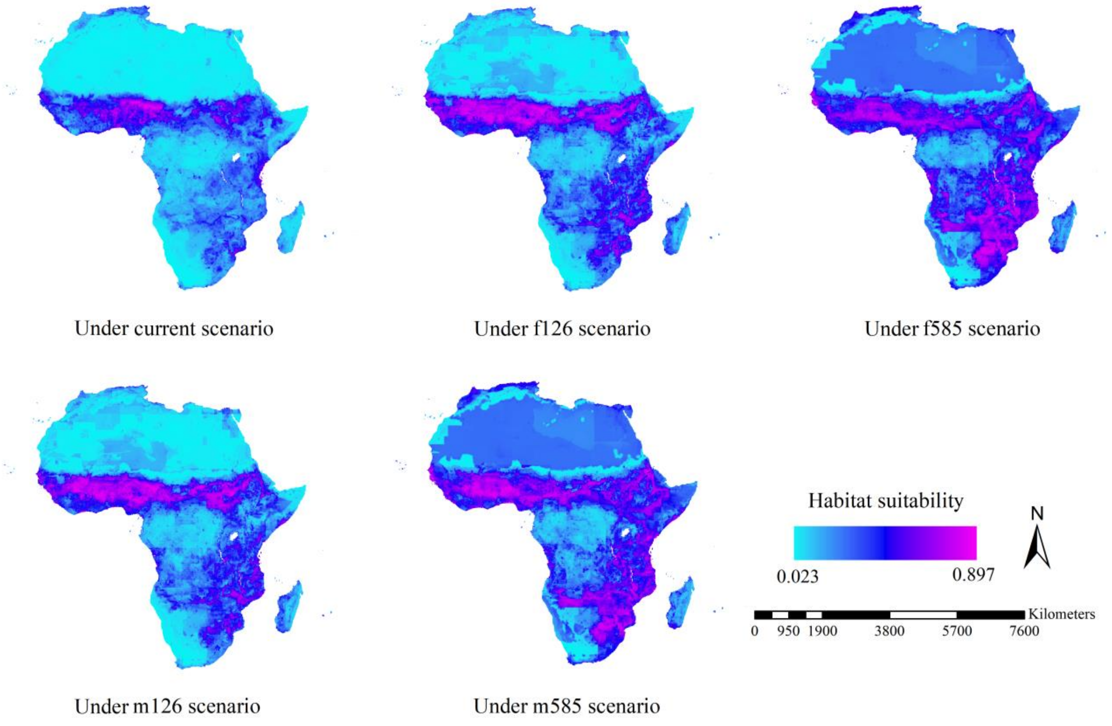

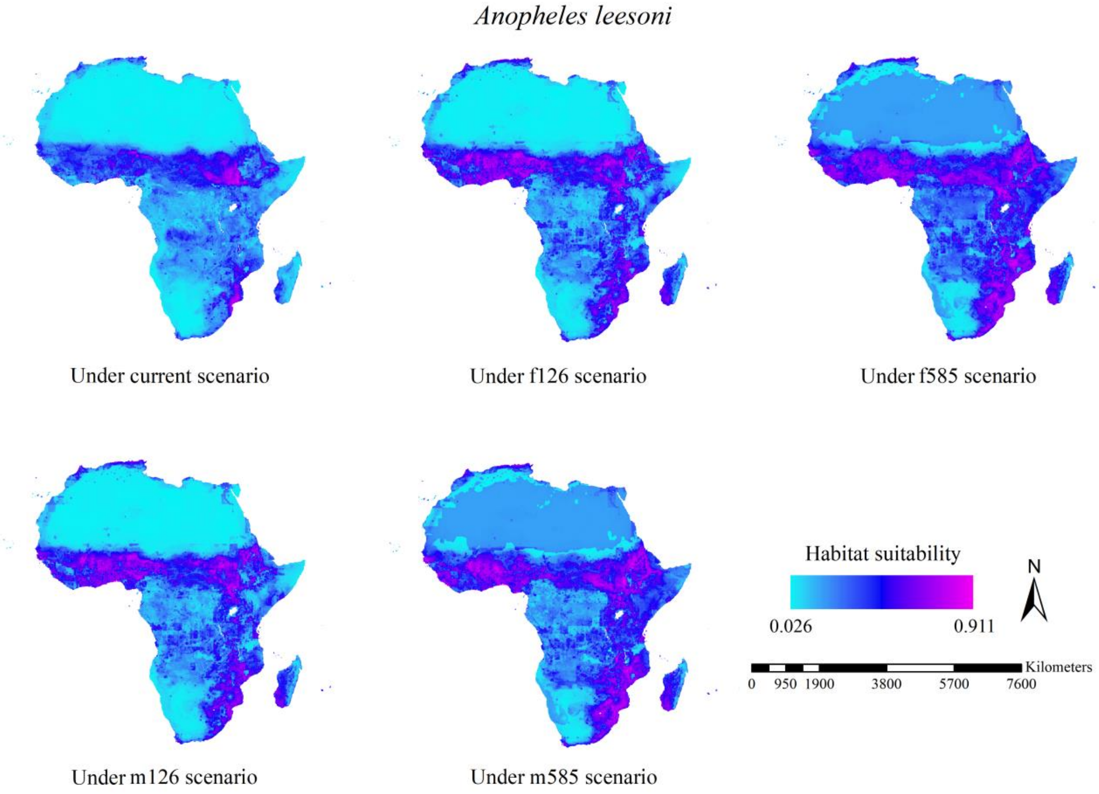

Anopheles leesoni
Under current scenario
Under f126 scenario
Under f585 scenario
Under m126 scenario
Under m585 scenario
Habitat suitability
0.026
0.911
N
Kilometers
0
950
1900
3800
5700
7600

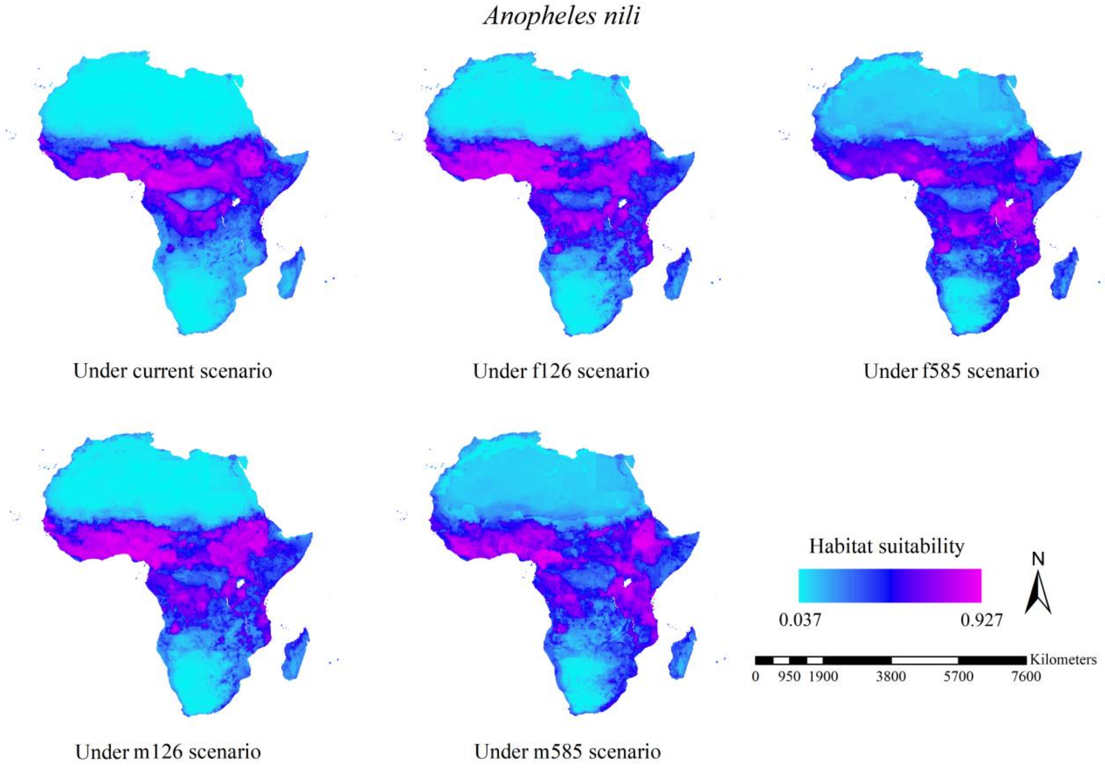
Anopheles nili
Under current scenario
Under f126 scenario
Under f585 scenario
Under m126 scenario
Under m585 scenario
Habitat suitability
0.037
0.927
N
Kilometers
0
950
1900
3800
5700
7600

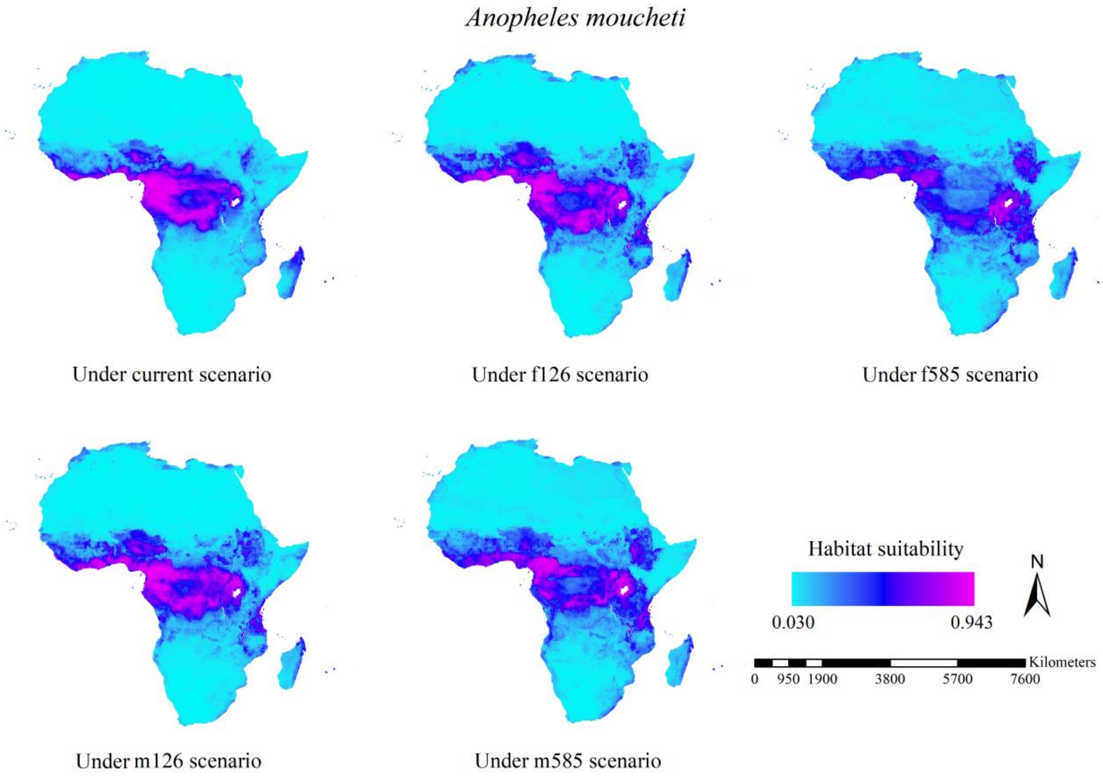
Anopheles moucheti
Under current scenario
Under f126 scenario
Under f585 scenario
Under m126 scenario
Under m585 scenario
Habitat suitability
0.030
0.943
N
Kilometers
0 950 1900 3800 5700 7600

Anopheles pharoensis

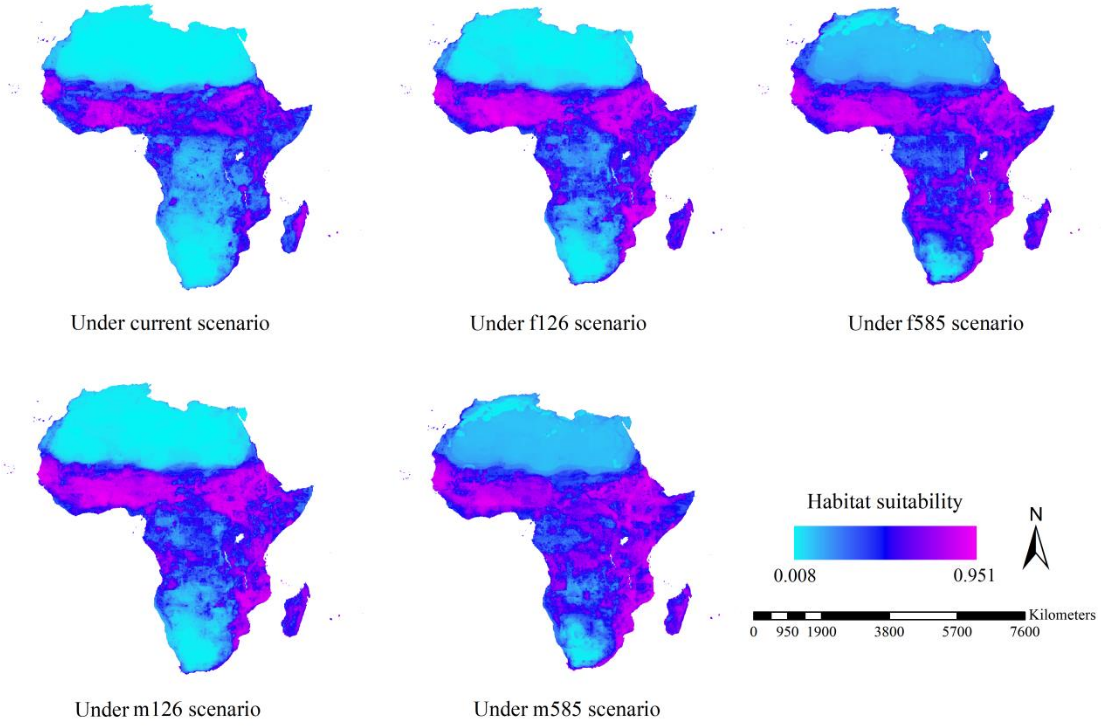

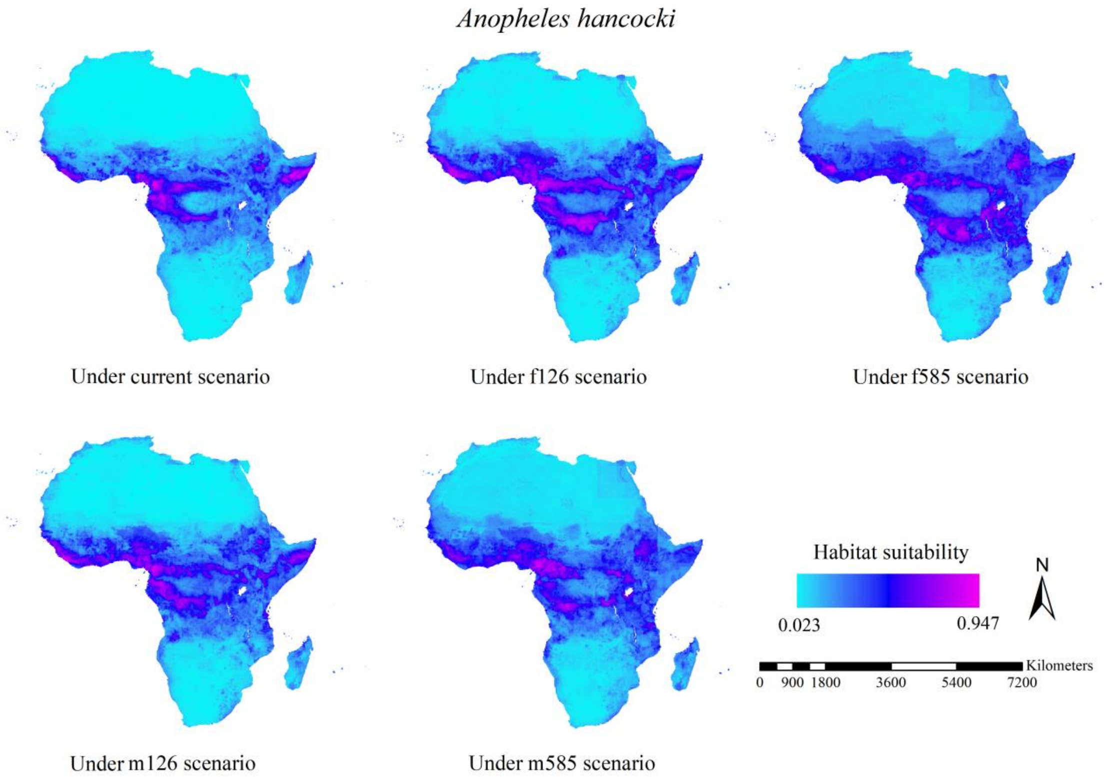

Anopheles hancocki
Under current scenario
Under f126 scenario
Under f585 scenario
Under m126 scenario
Under m585 scenario
Habitat suitability
0.023
0.947
N
Kilometers
0
900
1800
3600
5400
7200

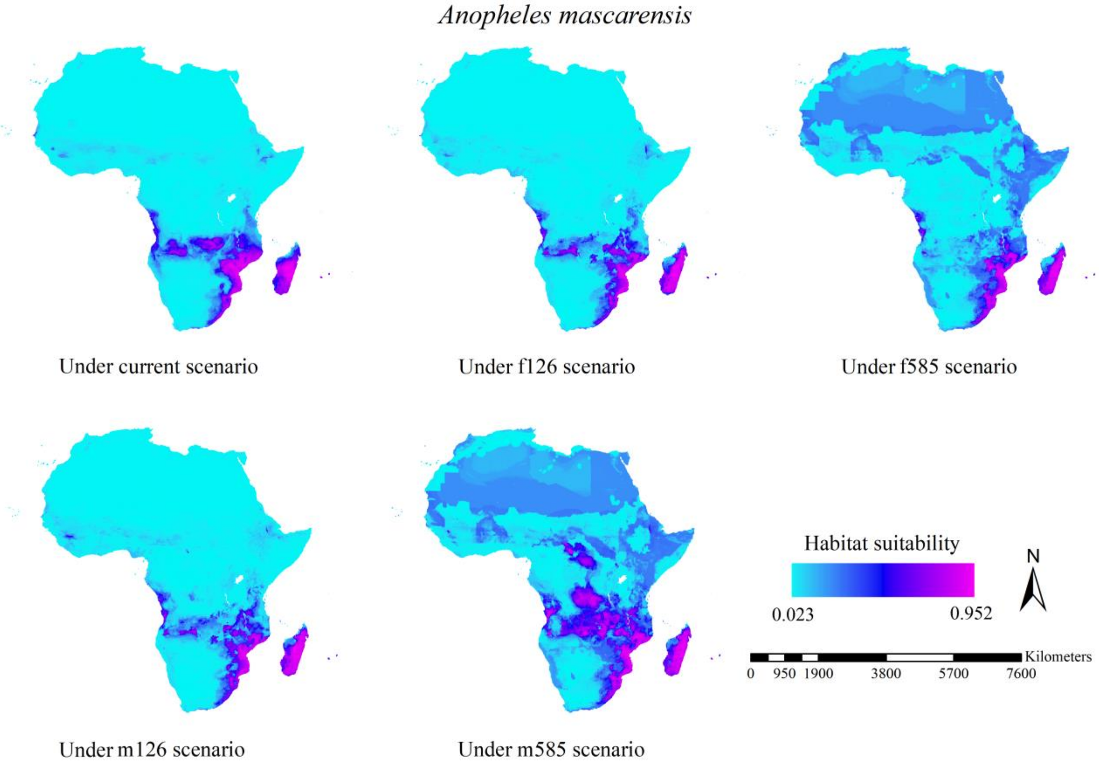
Anopheles mascarensis
Under current scenario
Under f126 scenario
Under f585 scenario
Under m126 scenario
Under m585 scenario
Habitat suitability
0.023
0.952
N
0 950 1900 3800 5700 7600
Kilometers

Anopheles marshalli

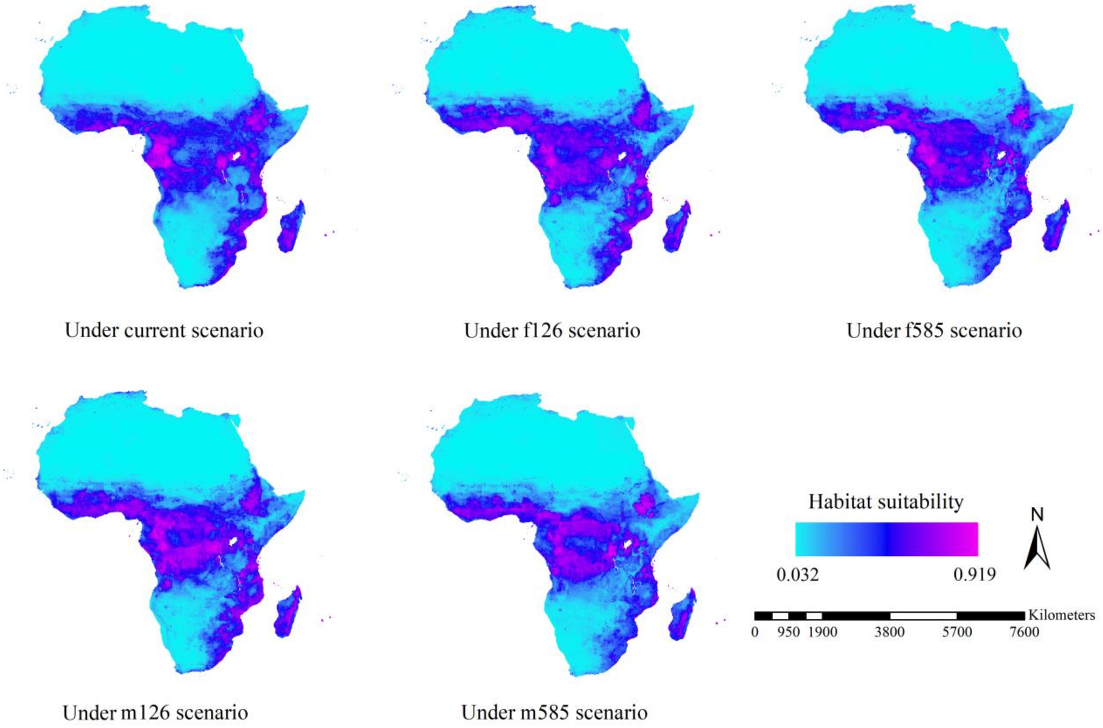

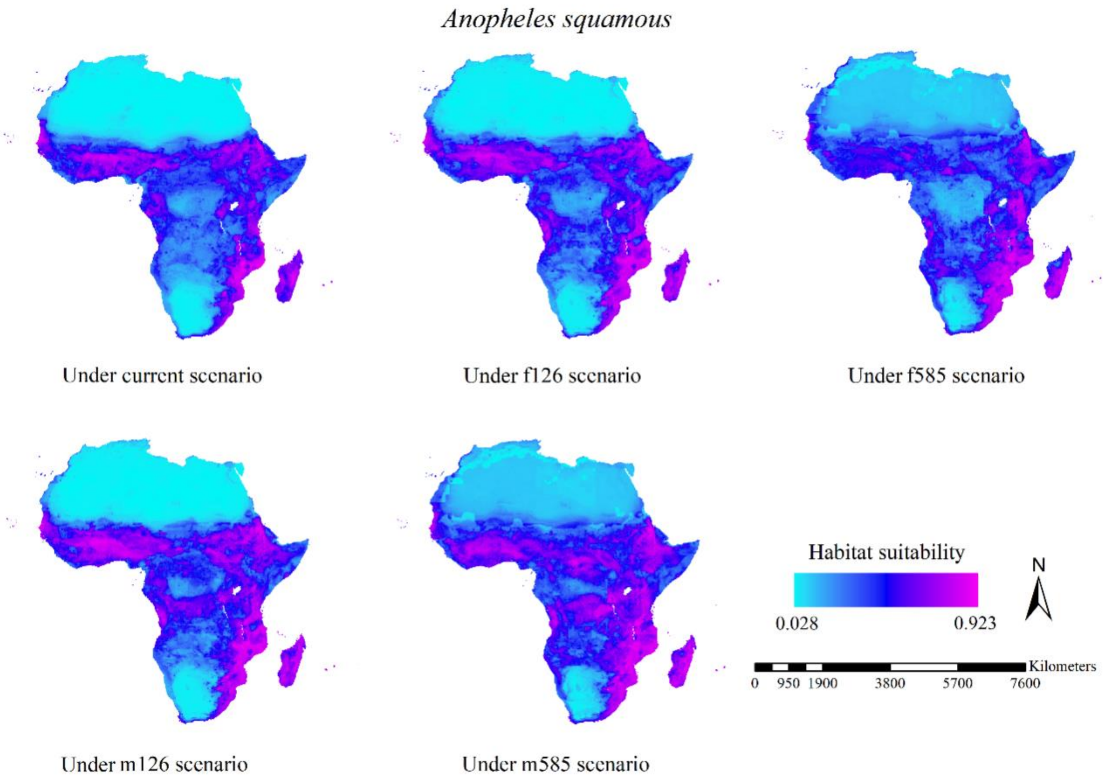
Anopheles squamous
Under current scenario
Under f126 scenario
Under f585 scenario
Under m126 scenario
Under m585 scenario
Habitat suitability
0.028
0.923
N
0 950 1900 3800 5700 7600
Kilometers

Anopheles wellcomei

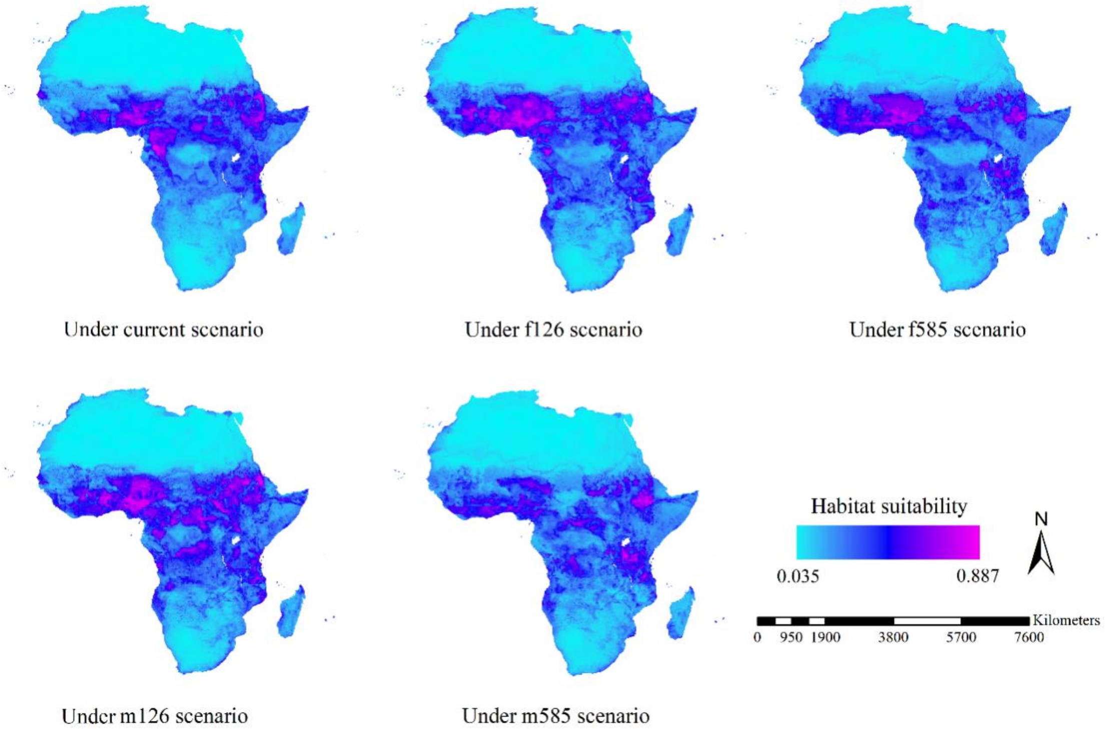

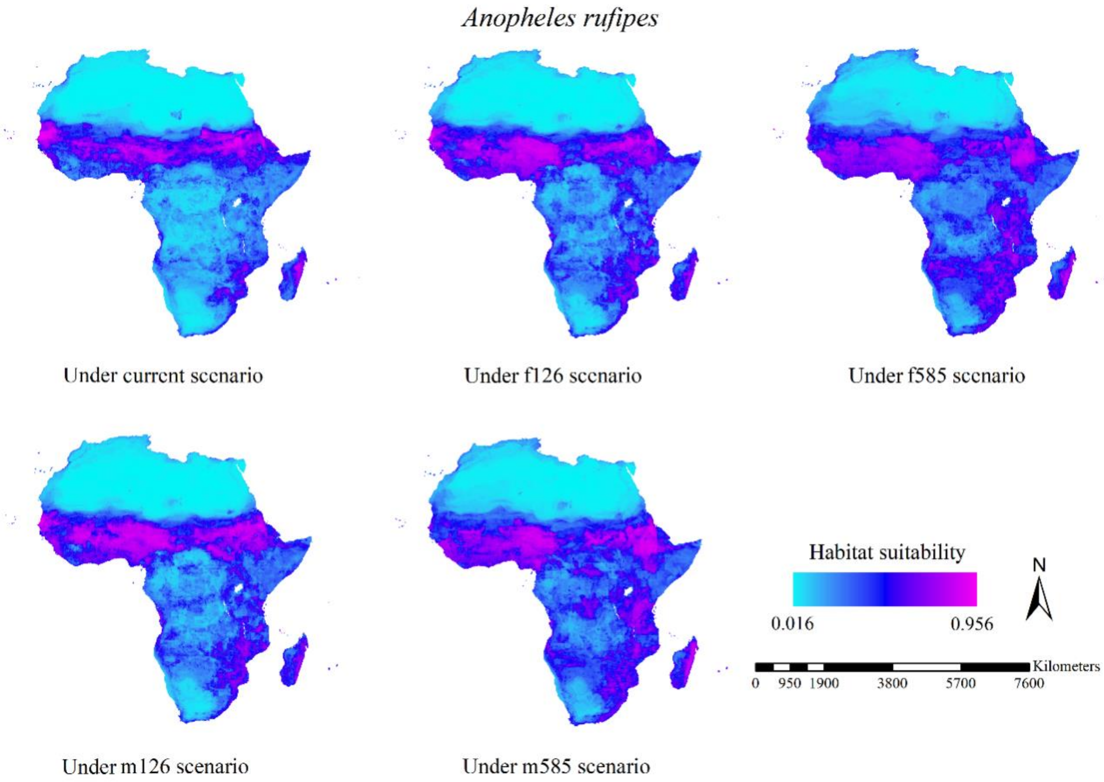

Anopheles rufipes
Under current scenario
Under f126 scenario
Under f585 scenario
Under m126 scenario
Under m585 scenario
Habitat suitability
0.016
0.956
N
0
950
1900
3800
5700
7600
Kilometers

Anopheles constani

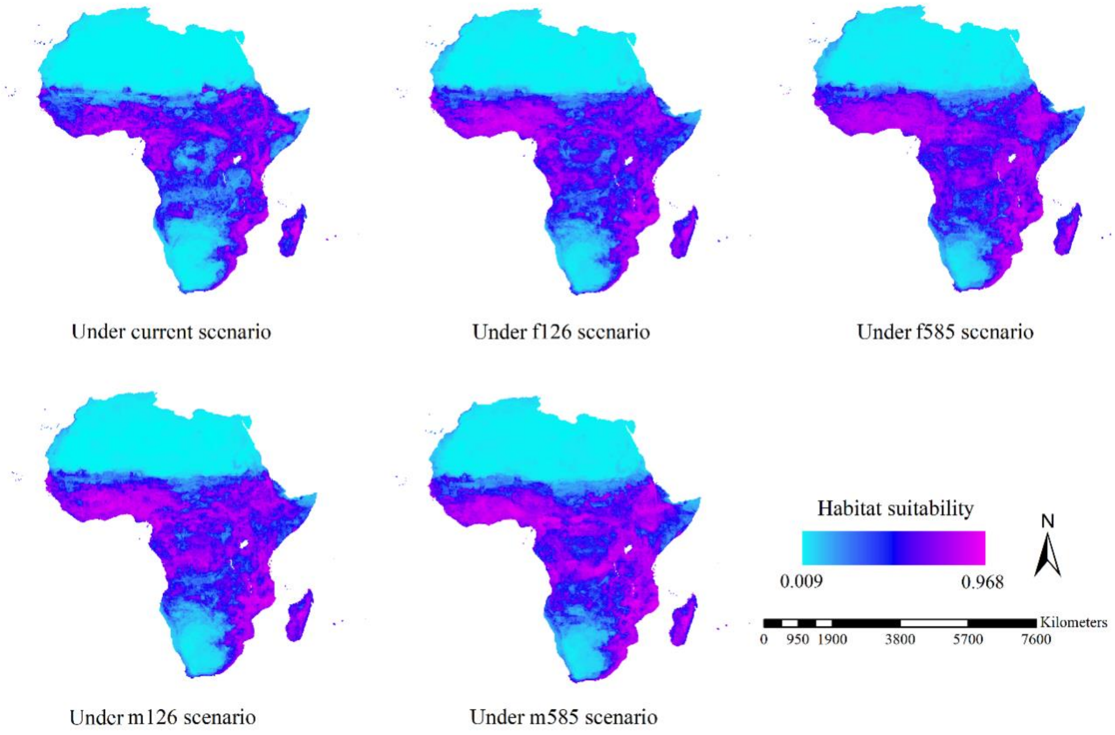

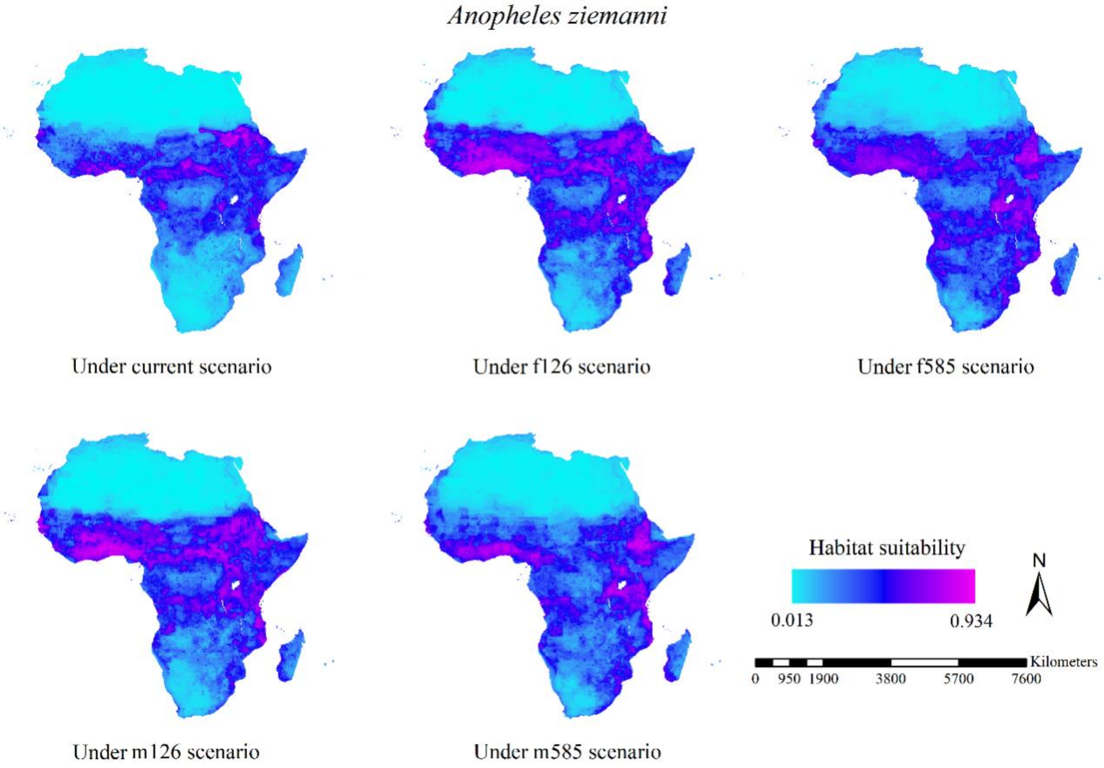
Anopheles ziemanni
Under current scenario
Under f126 scenario
Under f585 scenario
Under m126 scenario
Under m585 scenario
Habitat suitability
0.013
0.934
N
0
950
1900
3800
5700
7600
Kilometers

Anopheles paludis

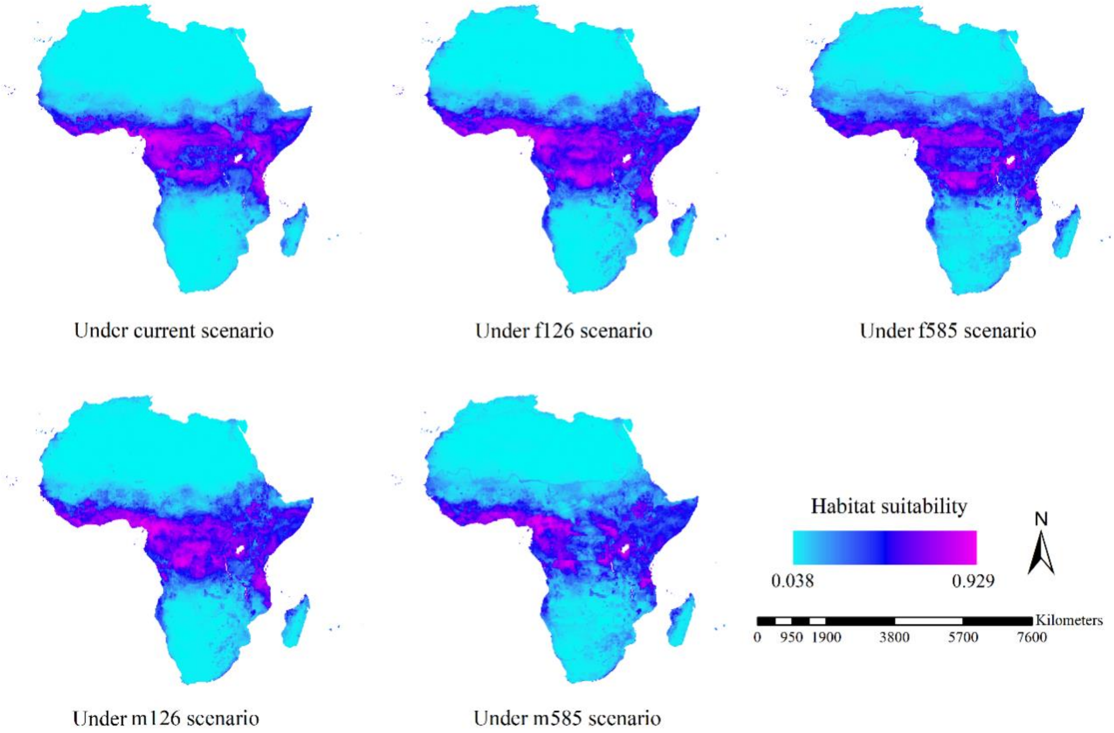

Anopheles coluzzii

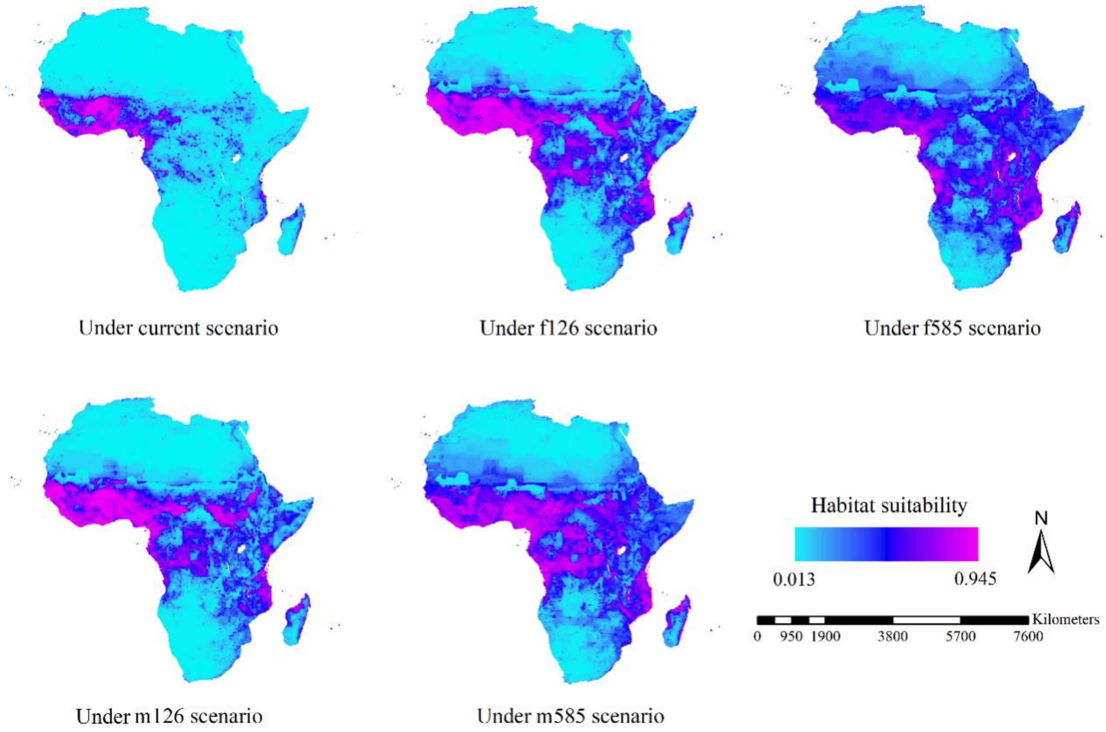

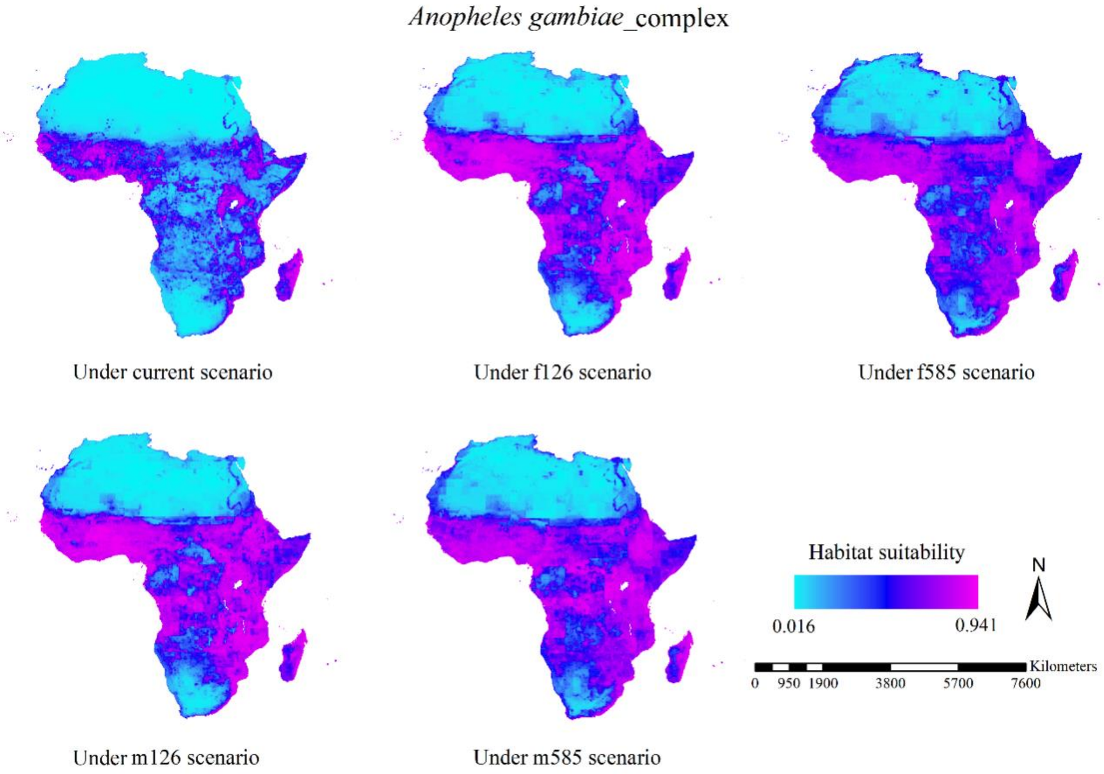
Anopheles gambiae_complex
Under current scenario
Under f126 scenario
Under f585 scenario
Under m126 scenario
Under m585 scenario
Habitat suitability
0.016
0.941
N
0
950
1900
3800
5700
7600
Kilometers
