# Supplementary material for: Range dynamics of Anopheles mosquitoes in Africa suggest a significant increase in the malaria transmission risk
Source: Ecol Evol. 2024 Jul 31;14(8):e70059. doi: 10.1002/ece3.70059 (PMC11289791; doi:10.1002/ece3.70059)
Supplement: Supplementary file 9 — Data S9: [file ECE3-14-e70059-s004.pdf]

S9 Potential ranges of 21 *Anopheles* species

Anopheles gambiae

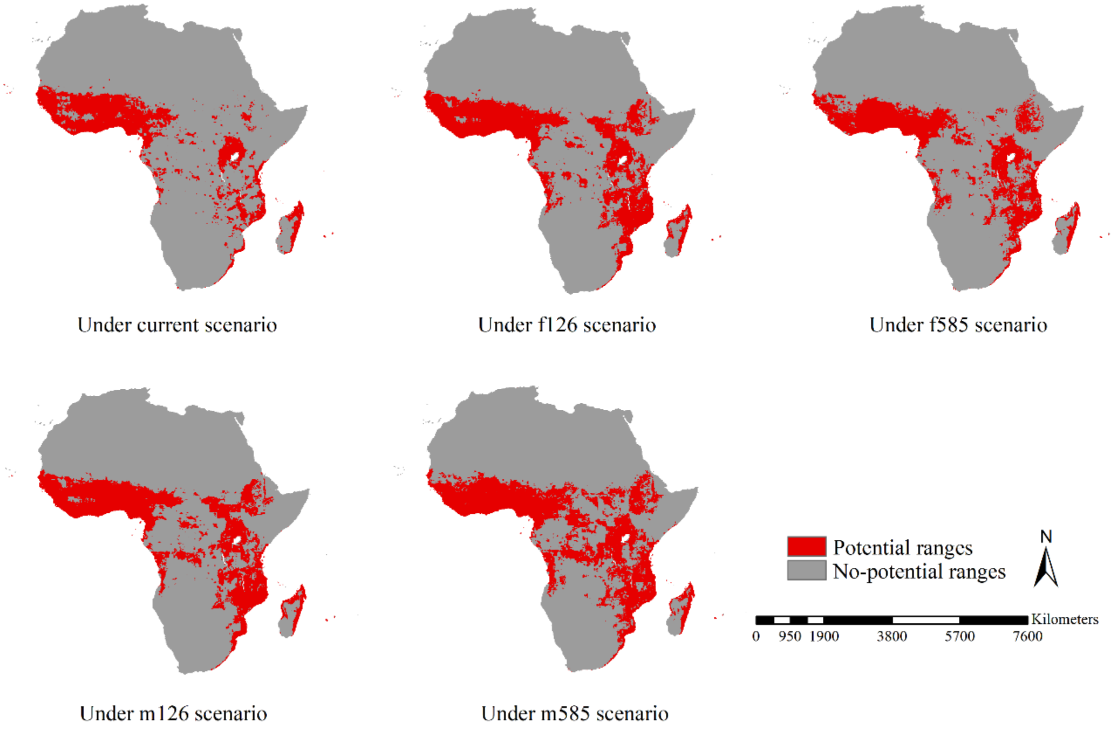

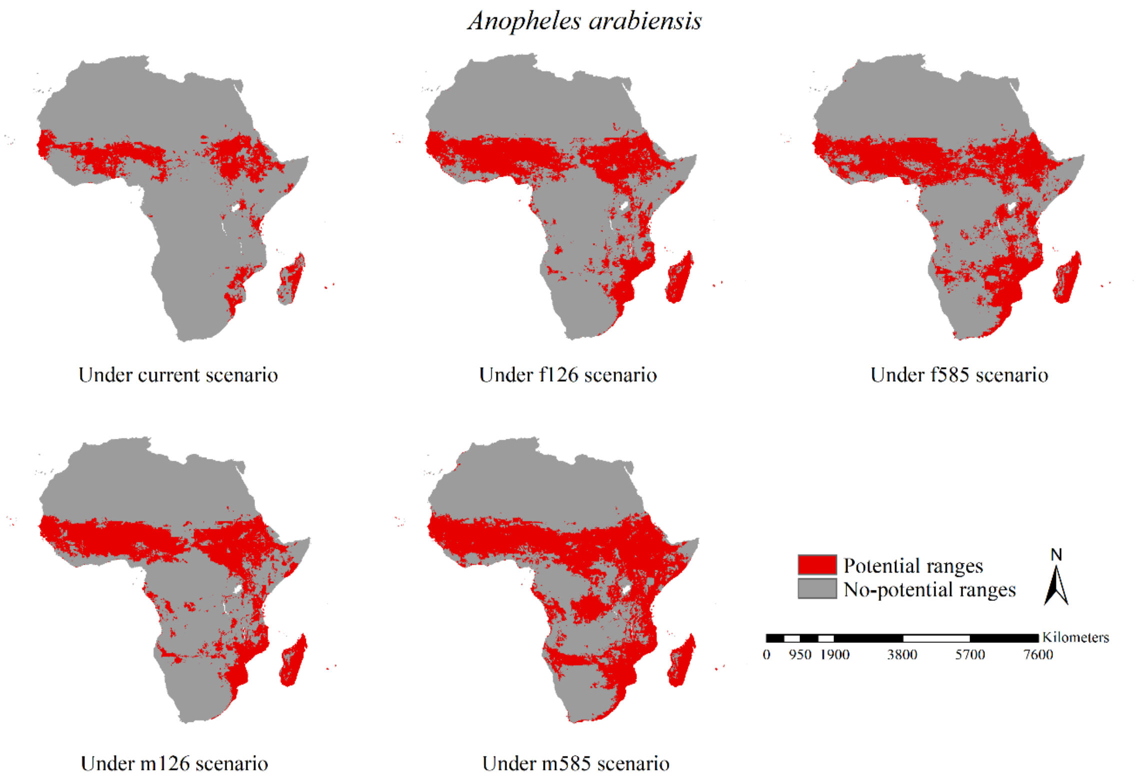

Anopheles arabiensis
Under current scenario
Under f126 scenario
Under f585 scenario
Under m126 scenario
Under m585 scenario
Potential ranges
No-potential ranges
N
Kilometers
0
950
1900
3800
5700
7600

Anopheles melas

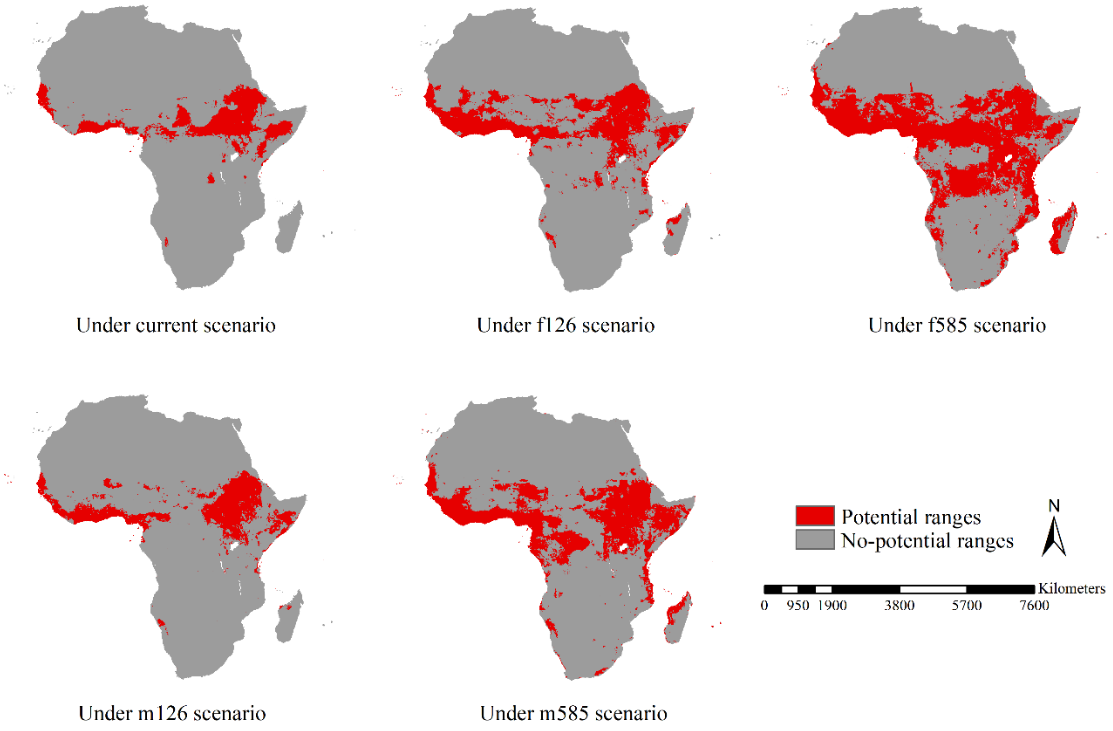

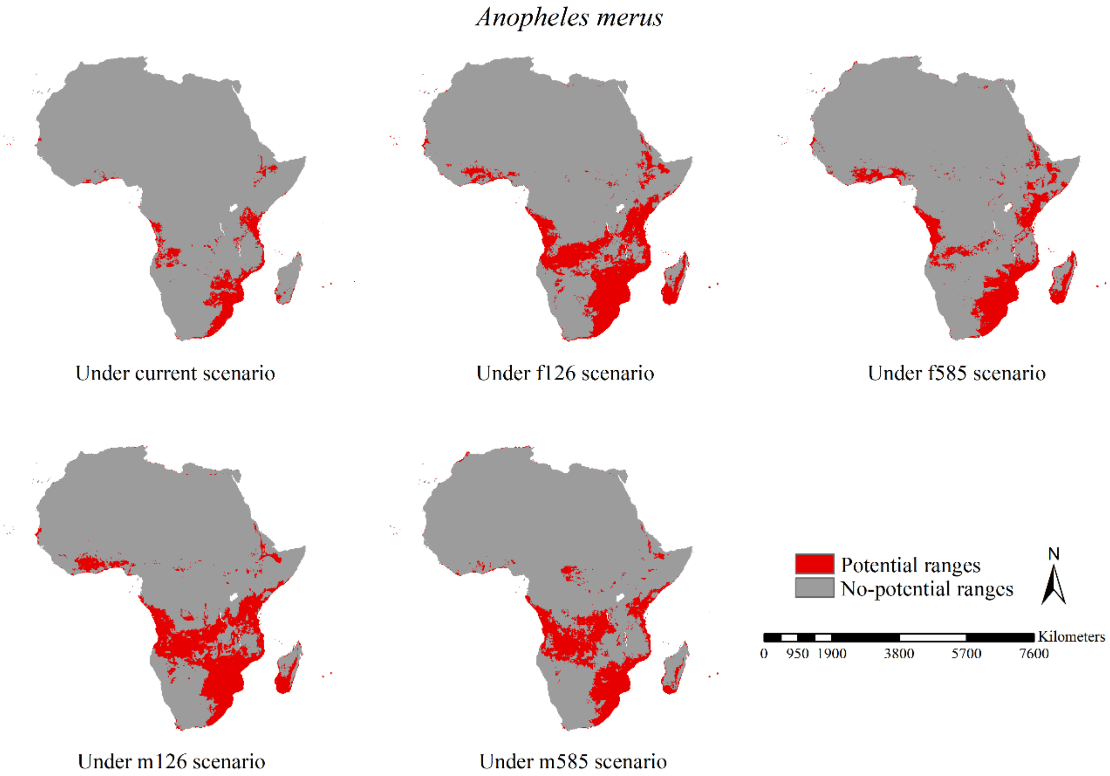
Anopheles merus
Under current scenario
Under f126 scenario
Under f585 scenario
Under m126 scenario
Under m585 scenario
Potential ranges
No-potential ranges
N
Kilometers
0
950
1900
3800
5700
7600

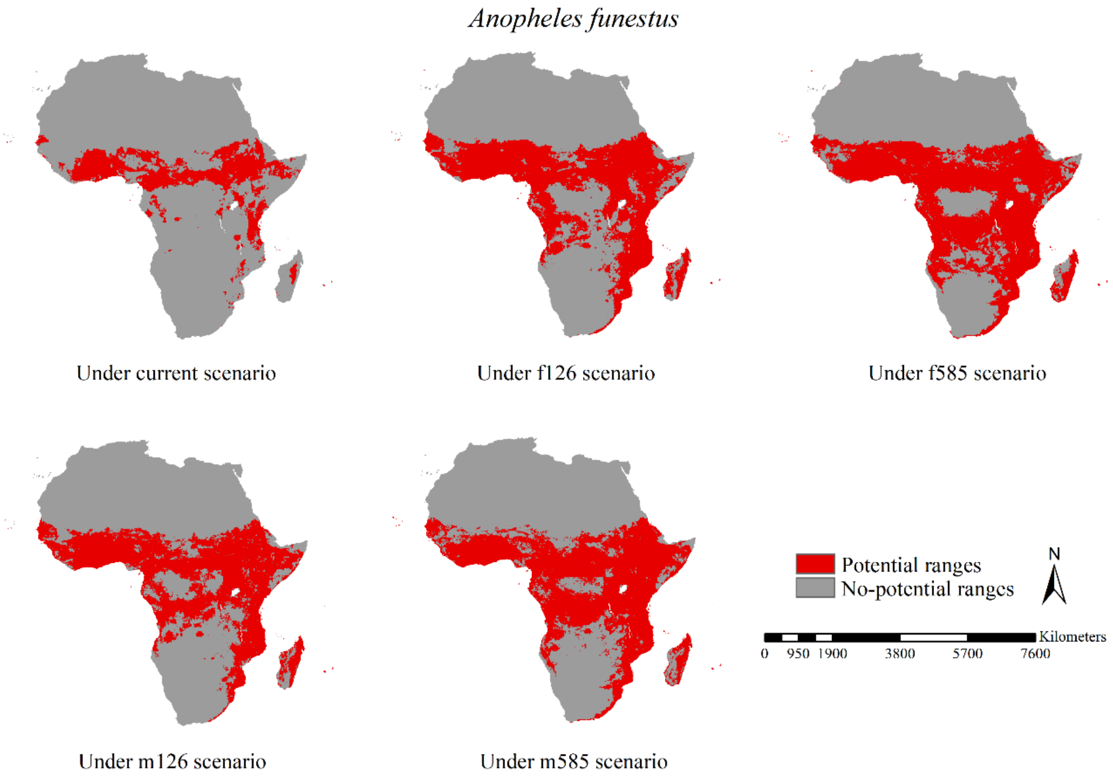
Anopheles funestus
Under current scenario
Under f126 scenario
Under f585 scenario
Under m126 scenario
Under m585 scenario
Potential ranges
No-potential ranges
N
Kilometers
0 950 1900 3800 5700 7600

Anopheles rivulorum

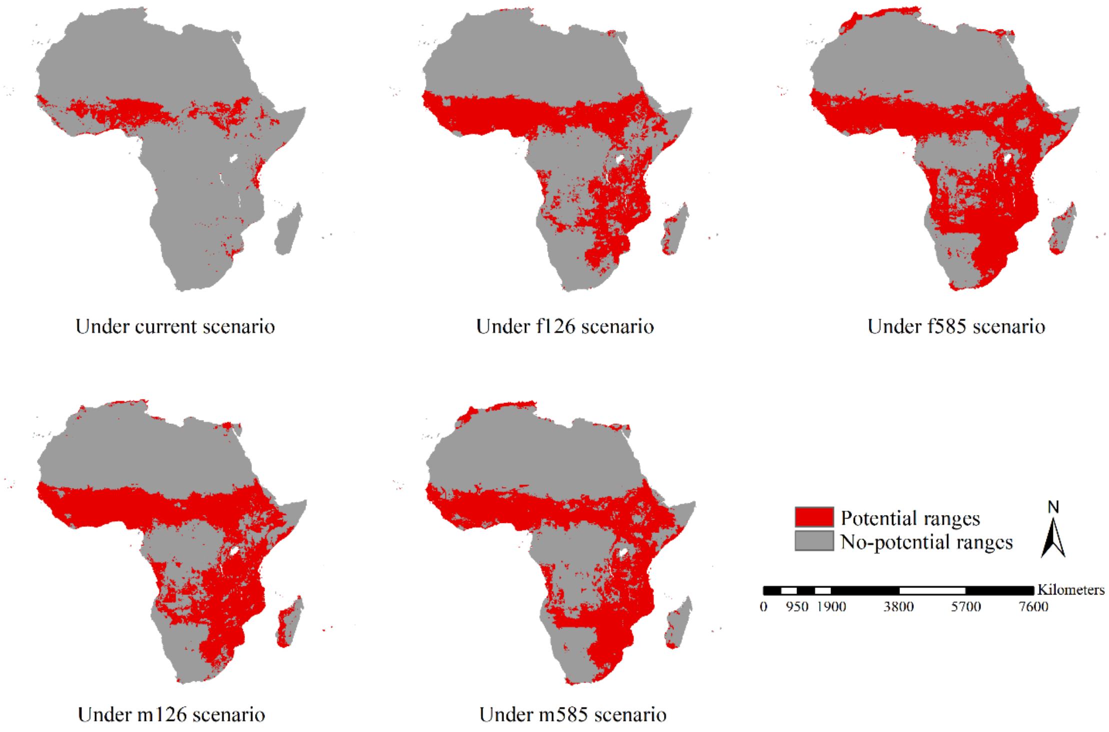

Anopheles leesoni

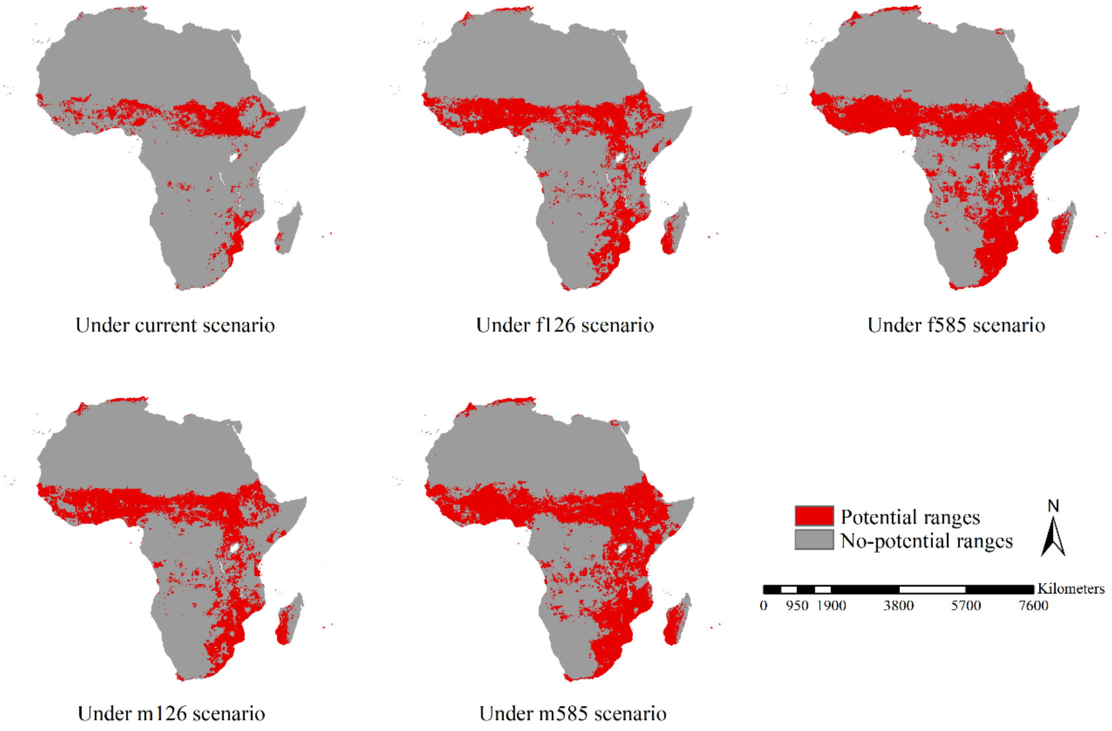

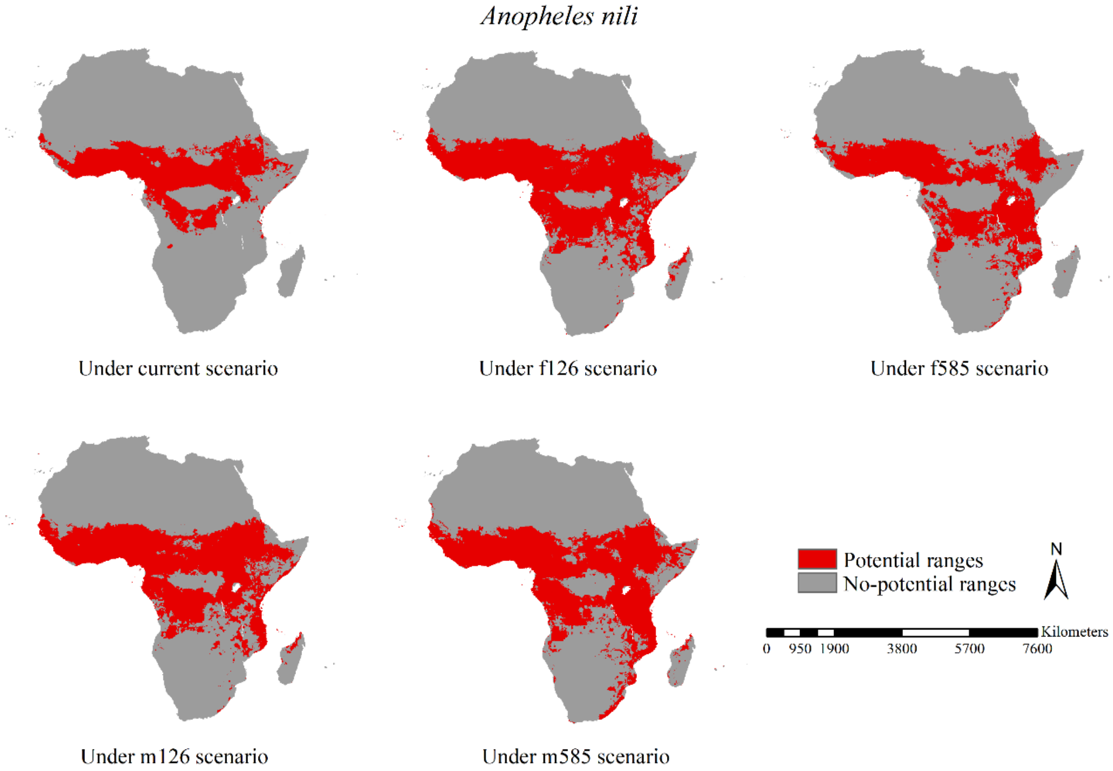
Anopheles nili
Under current scenario
Under f126 scenario
Under f585 scenario
Under m126 scenario
Under m585 scenario
Potential ranges
No-potential ranges
N
Kilometers
0 950 1900 3800 5700 7600

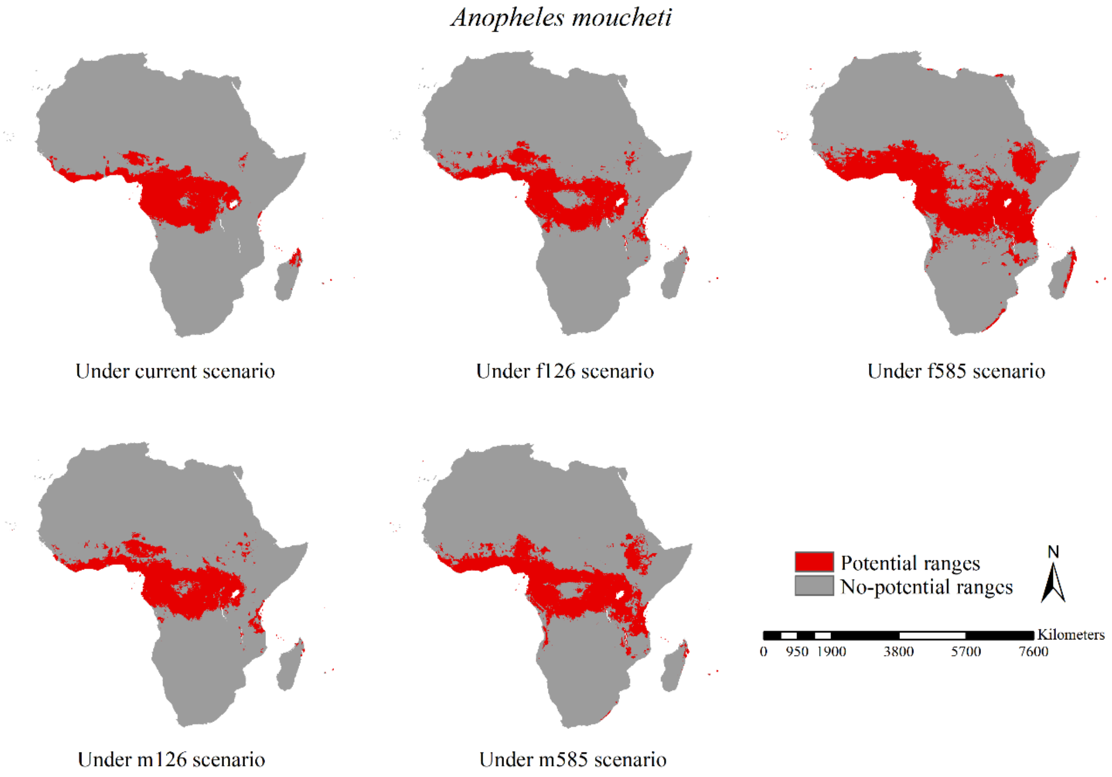
Anopheles moucheti
Under current scenario
Under f126 scenario
Under f585 scenario
Under m126 scenario
Under m585 scenario
Potential ranges
No-potential ranges
N
Kilometers
0 950 1900 3800 5700 7600

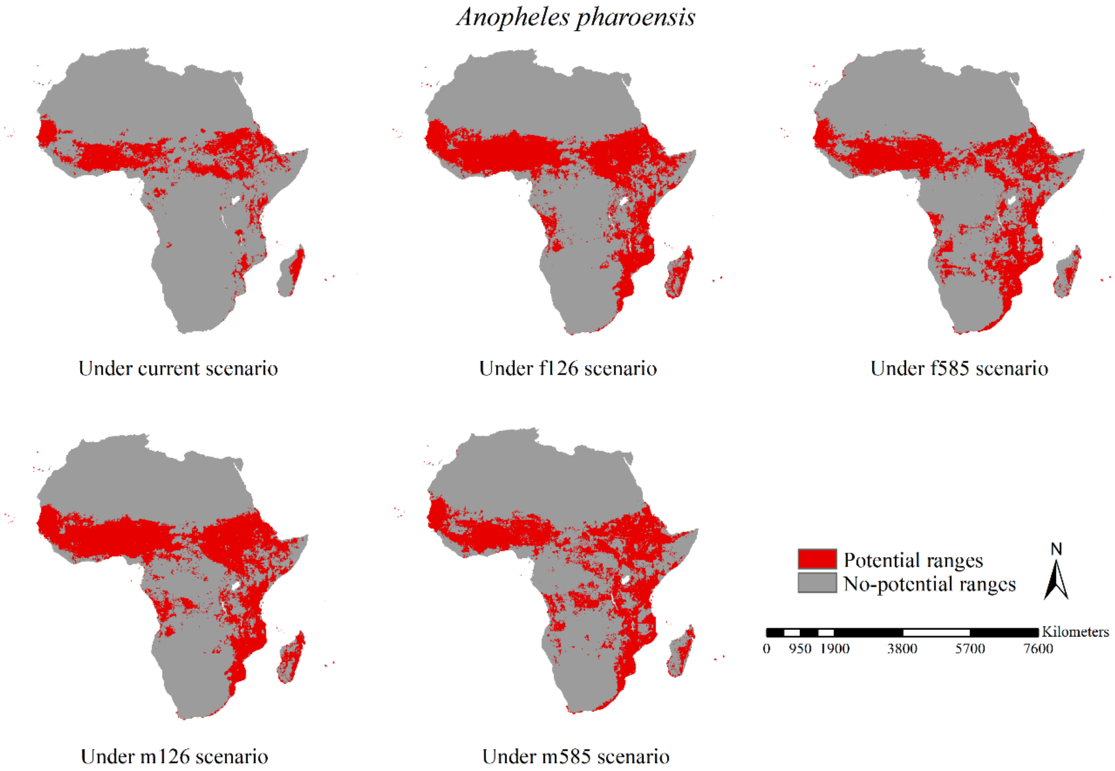
Anopheles pharoensis
Under current scenario
Under f126 scenario
Under f585 scenario
Under m126 scenario
Under m585 scenario
Potential ranges
No-potential ranges
N
Kilometers
0 950 1900 3800 5700 7600

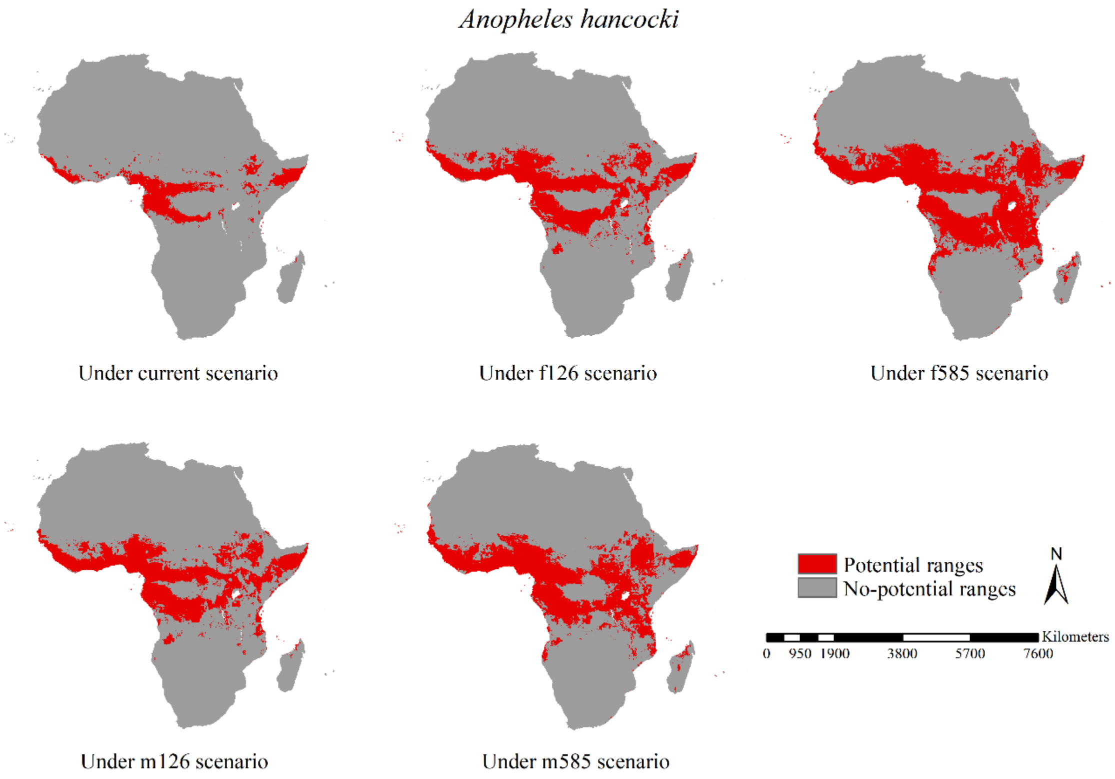
Anopheles hancocki
Under current scenario
Under f126 scenario
Under f585 scenario
Under m126 scenario
Under m585 scenario
Potential ranges
No-potential ranges
N
Kilometers
0
950
1900
3800
5700
7600

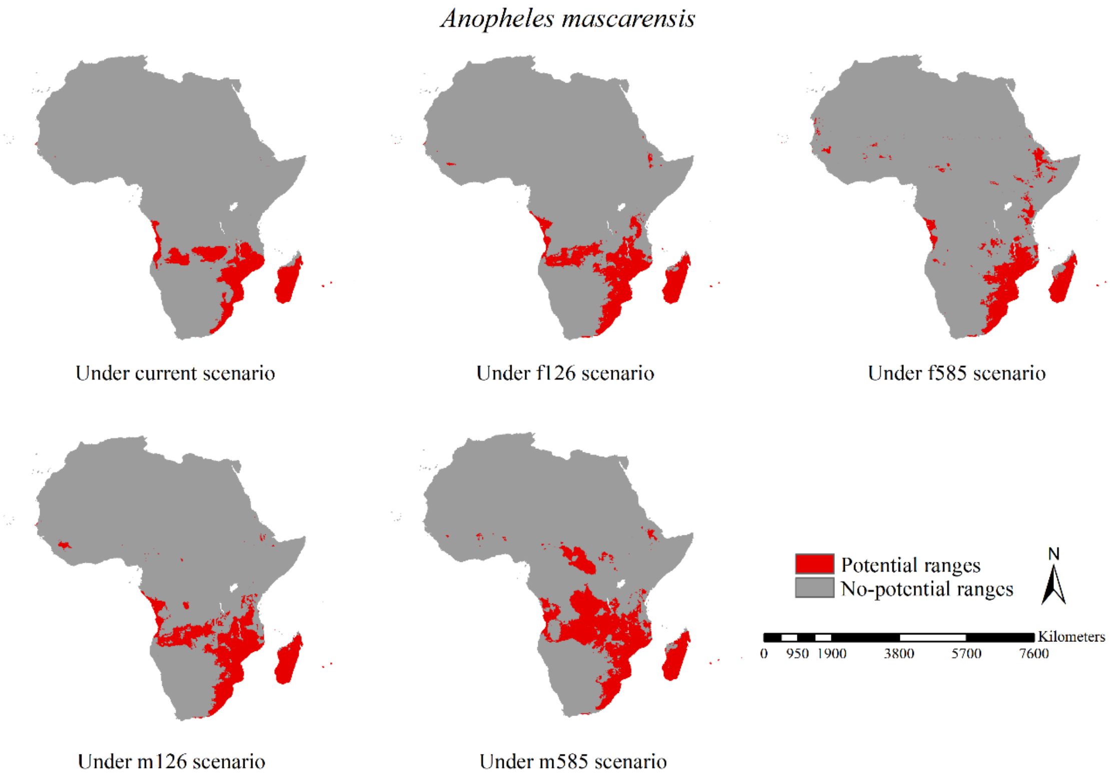
Anopheles mascarensis
Under current scenario
Under f126 scenario
Under f585 scenario
Under m126 scenario
Under m585 scenario
Potential ranges
No-potential ranges
N
Kilometers
0 950 1900 3800 5700 7600

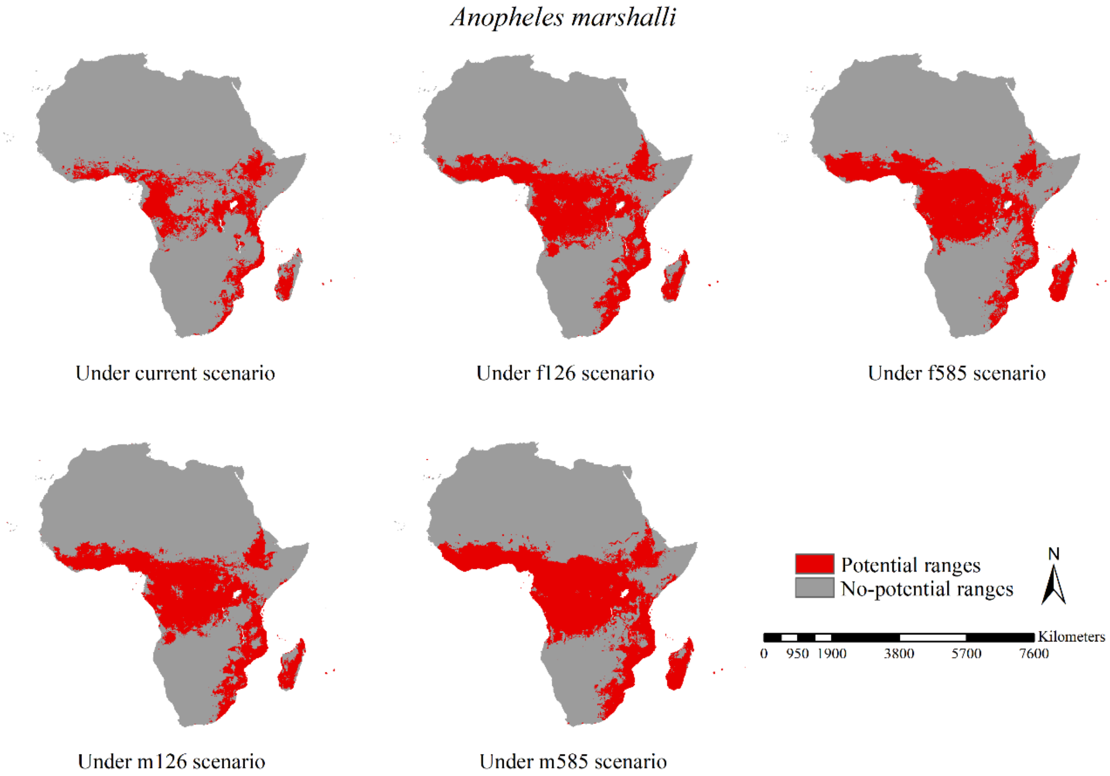
Anopheles marshalli
Under current scenario
Under f126 scenario
Under f585 scenario
Under m126 scenario
Under m585 scenario
Potential ranges
No-potential ranges
N
Kilometers
0
950
1900
3800
5700
7600

Anopheles squamous

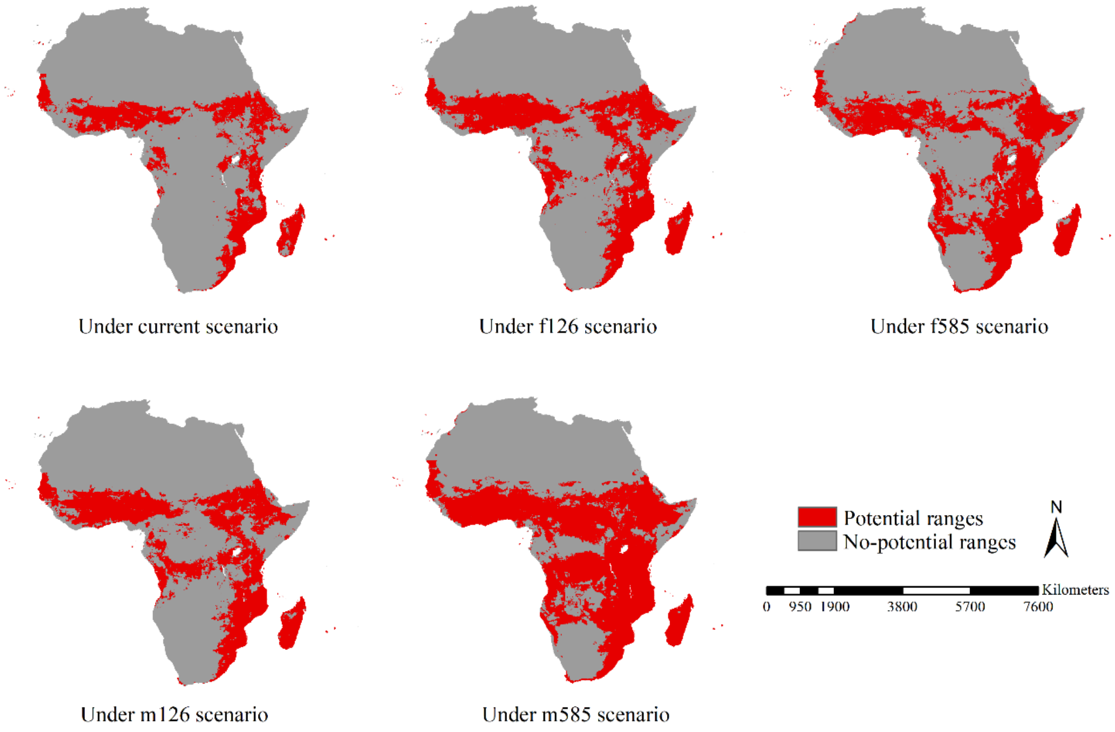

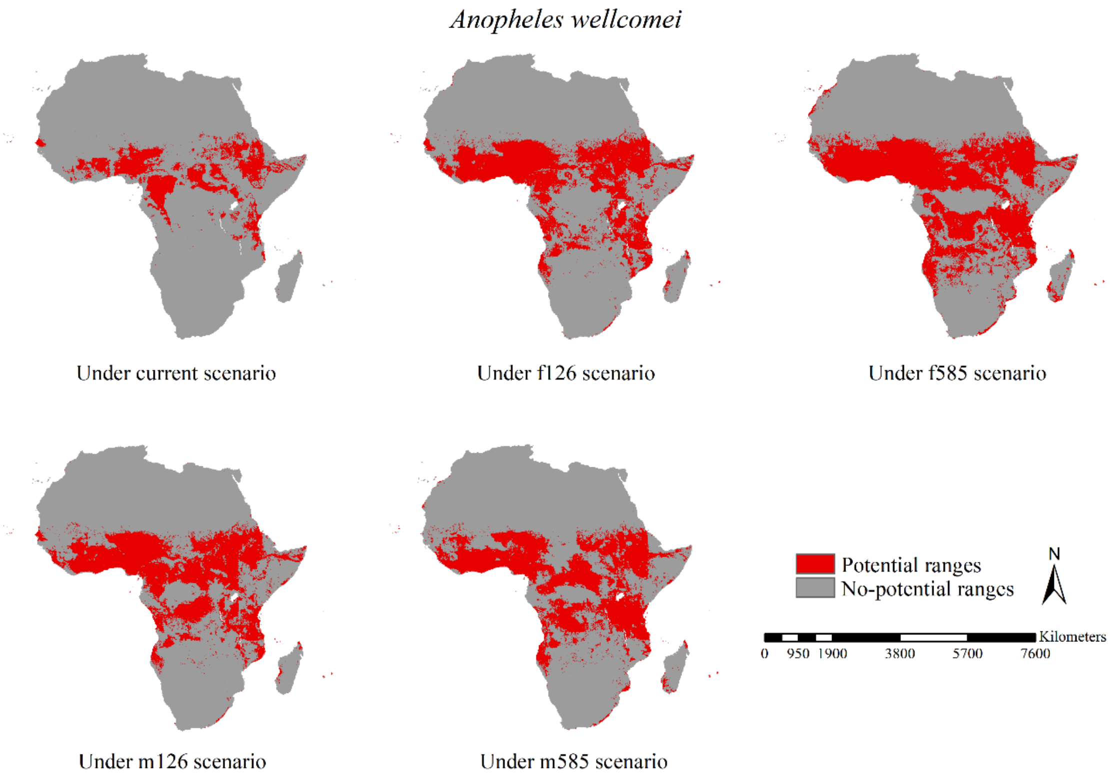
Anopheles wellcomei
Under current scenario
Under f126 scenario
Under f585 scenario
Under m126 scenario
Under m585 scenario
Potential ranges
No-potential ranges
N
Kilometers
0 950 1900 3800 5700 7600

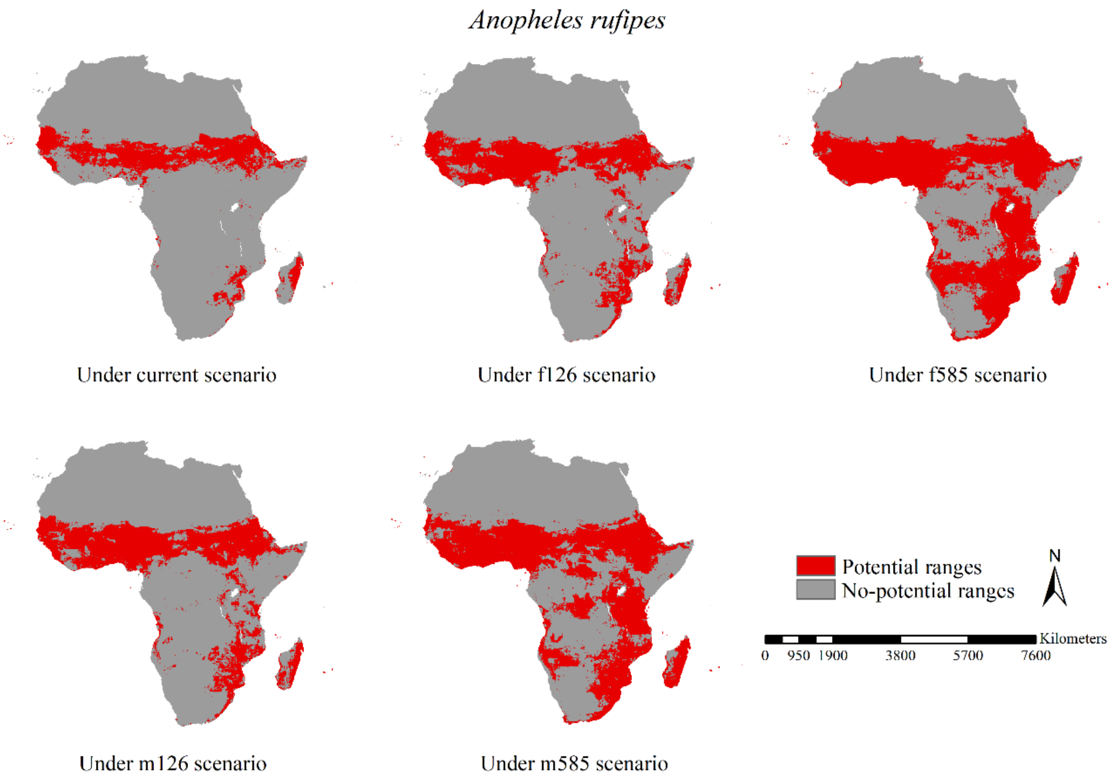
Anopheles rufipes
Under current scenario
Under f126 scenario
Under f585 scenario
Under m126 scenario
Under m585 scenario
Potential ranges
No-potential ranges
N
Kilometers
0 950 1900 3800 5700 7600

Anopheles constani

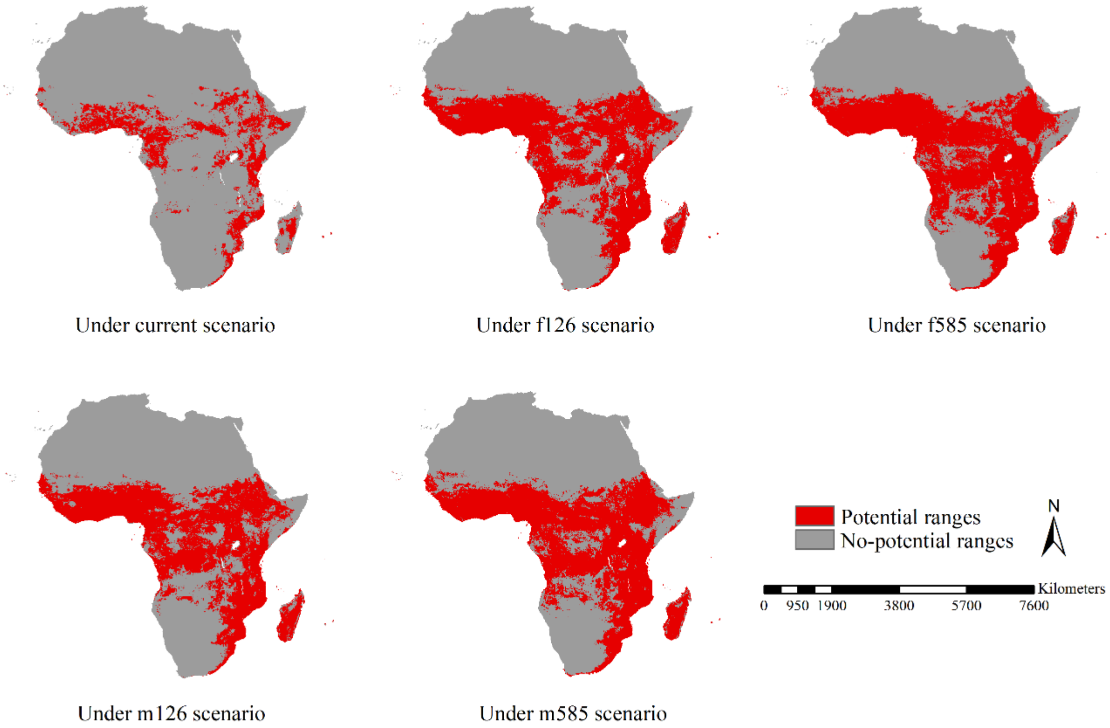

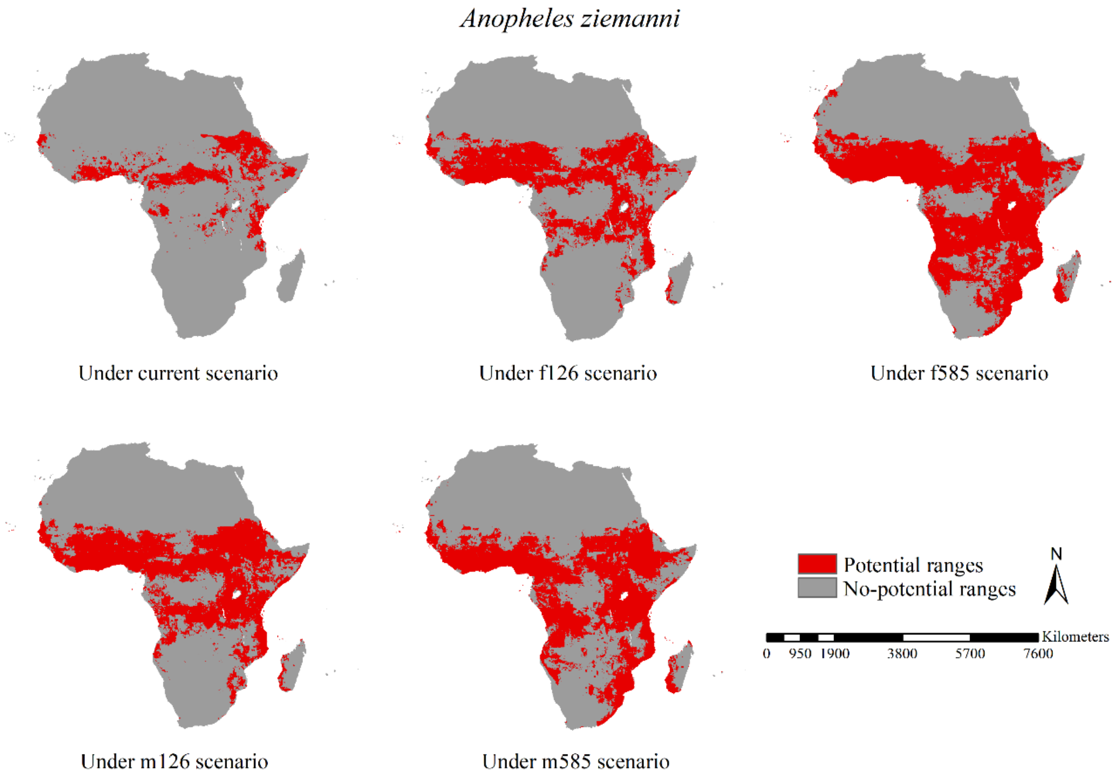
Anopheles ziemanni
Under current scenario
Under f126 scenario
Under f585 scenario
Under m126 scenario
Under m585 scenario
Potential ranges
No-potential ranges
N
Kilometers
0 950 1900 3800 5700 7600

Anopheles paludis

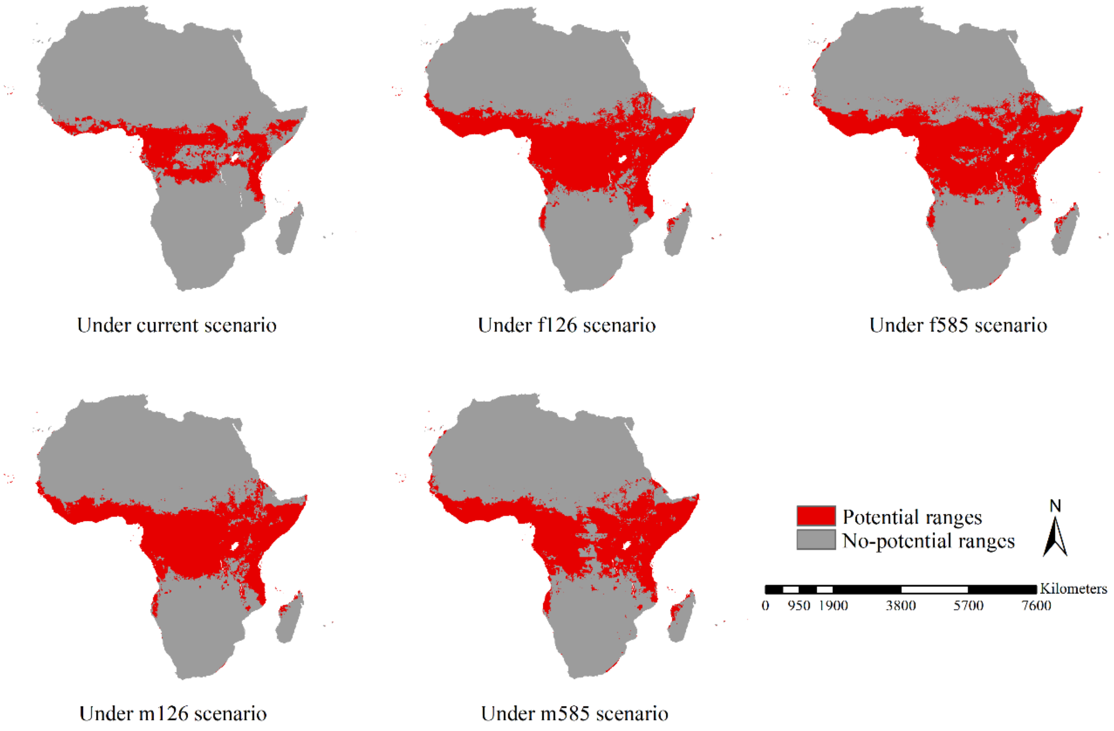

Anopheles coluzzii

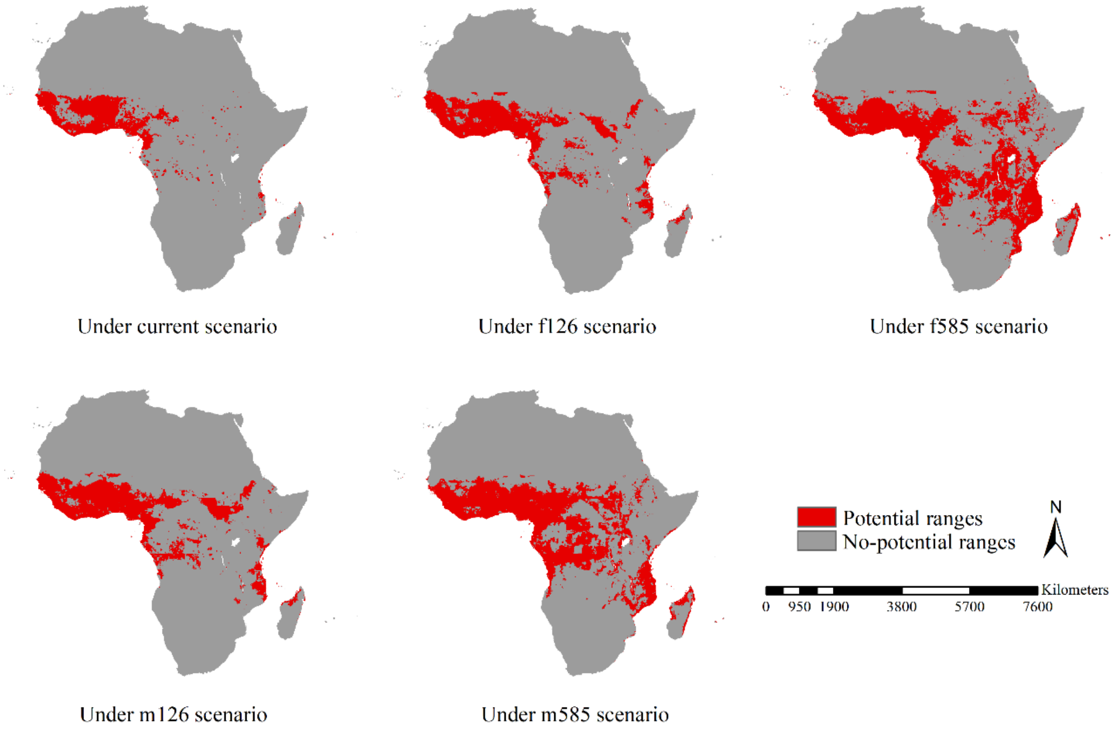

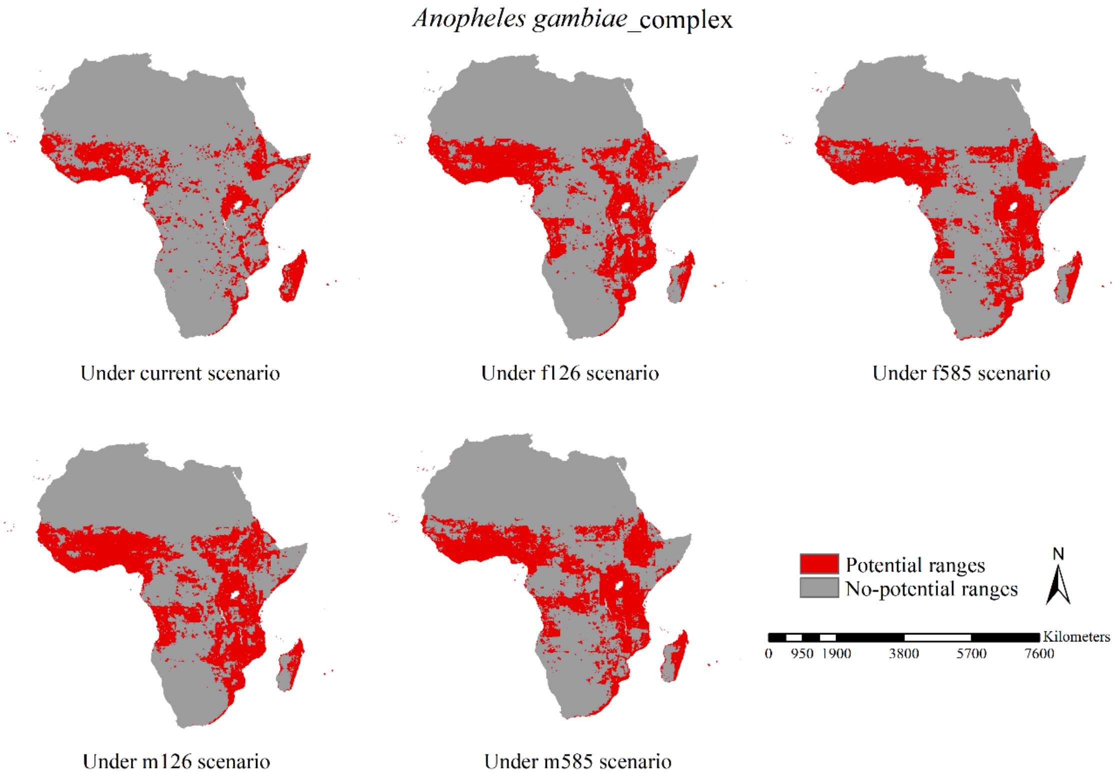
Anopheles gambiae_complex
Under current scenario
Under f126 scenario
Under f585 scenario
Under m126 scenario
Under m585 scenario
Potential ranges
No-potential ranges
N
Kilometers
0 950 1900 3800 5700 7600
